# Food diversification as an adaptation strategy to climate change: Habitat suitability for wild and cultivated food plants in the Brazilian Semiarid

**DOI:** 10.1007/s13280-026-02374-2

**Published:** 2026-04-04

**Authors:** Amanda Stefanie Sérgio da Silva, Xavier Arnan, Patrícia Muniz de Medeiros

**Affiliations:** 1 Postgraduate Program in Ethnobiology and Nature Conservation, Federal Rural University of Pernambuco, Dom Manoel de Medeiros, s/n, Dois Irmãos, Recife, PE 52171-900 Brazil; 2 Mamirauá Institute for Sustainable Development, Instituto de Desenvolvimento Sustentável Mamirauá, Estrada do Bexiga, 2.584, Fonte Boa, Tefé, AM 69553-225 Brazil; 3 University of Pernambuco, Campus Garanhuns, Rua Cap. Pedro Rodrigues, 105, São José, Garanhuns, PE 55294-902 Brazil; 4 Federal University of Alagoas, Universidade Federal de Alagoas, Centro de Ciências Agrárias, Campus of Engineering and Agricultural Sciences, BR 104, Km 85, s/n, Rio Largo, AL 57100-000 Brazil

**Keywords:** Climate risk, Family farming, Food and nutrition security, Food systems, Food unavailability, Species distribution modeling

## Abstract

This study aimed to assess the potential effect of climate change on habitat suitability for wild and cultivated food plants under future scenarios. Another aim was to determine whether food diversification can reduce the risk of food unavailability. Habitat suitability models were constructed for wild and cultivated species of economic and dietary importance and projected for future climate scenarios (2041–2060) using the Brazilian Semiarid as a case study. Variations in habitat suitability and species number were estimated for the species groups. The results showed that climate change can reduce habitat suitability for food plants, with a greater potential reduction for cultivated plants. The potential risk of species unavailability differed between groups. High-risk areas (species loss > 50%) were larger for cultivated species than for wild and cultivated species together. Thus, food diversification represents a sustainable solution to promote adaptation to climate change and maintain food security in the future.

**Graphical abstract:**

**Figure:**
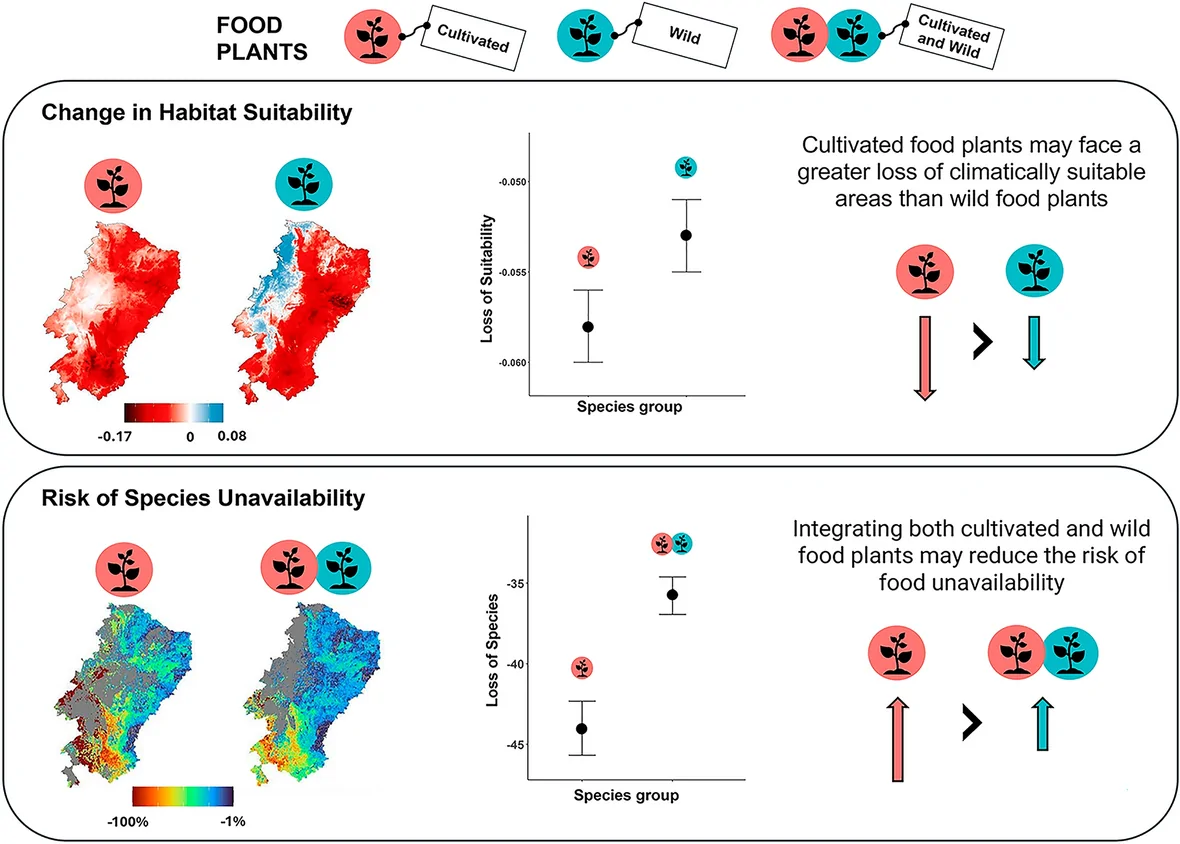

**Supplementary Information:**

The online version contains supplementary material available at https://doi.org/10.1007/s13280-026-02374-2.

## Introduction

Climate change poses risks to natural ecosystems and human well-being (Pecl et al. 2017; IPCC 2023). It can alter ecosystem functions and processes, threatening species survival (Scheffers et al. 2016) and exerting negative effects on agricultural activity and food production (Moore et al. 2017; Zhu et al. 2022). Studies have demonstrated that suitable areas for agriculture and agricultural productivity are shrinking worldwide, particularly in tropical regions and developing countries (Challinor et al. 2014; Zabel et al. 2014; Zhu et al. 2022). Reduced crop yields could lead to higher food prices (Stevanović et al. 2016; Peña-Lévano et al. 2019), as well as greater use of agricultural inputs and technologies (Caetano et al. 2018). Consequently, climate change can impact food supply and compromise food and nutrition security (Mbow et al. 2022; Zhu et al. 2022).

Cultivated plants have been selected by human populations through domestication to adapt to specific environmental conditions, resulting in changes (increases or reductions) in the genetic variability of species (Lippmann et al. 2019). However, even though certain cultivated species exhibit considerable genetic variation, modern agriculture tends to rely on a small portion of cultivars, focusing on those that provide the greatest yields (Bita and Gerats 2013; Khoury et al. 2022). Low genetic variability may reduce the compatibility between plant species and the environment, rendering them more vulnerable to extreme climatic events, such as droughts, floods, and sudden temperature changes (Bita and Gerats 2013; Gao et al. 2023). By contrast, uncultivated (wild) plants typically show greater genetic variability and good adaptability to a broader range of environmental conditions. Such characteristics enable them to resist and adapt to climate change (Hoffmann and Sgrò 2011). It is therefore crucial to diversify productive landscapes and species within food systems (Gergel et al. 2020; Powell et al. 2023).

Food diversification refers to incorporating a wide variety of foods in the diet, including different food groups, such as animal proteins, fruits, vegetables, and grains (FAO 2019). In an agricultural context, food diversification consists of introducing and cultivating different species in production systems (Vernooy 2022). Diversification strategies can make agricultural systems more sustainable and resilient to pests, diseases, and environmental changes (Gepts 2023). From a broader perspective, food diversification also includes the incorporation of wild species into the diet, providing additional sources of nutrients (Powell et al. 2015). Wild plants can also be introduced to food systems to increase the resilience of plant communities along environmental gradients and promote species survival and availability in a context of climate change (Hoffmann and Sgrò 2011; Fromentin et al. 2023; Powell et al. 2023).

Food systems have become a central axis of international policy frameworks aimed at climate change mitigation and adaptation. The IPCC explicitly recognizes that agricultural diversification, agroecology, and the conservation of agrobiodiversity are low-cost, high co-benefit strategies to reduce emissions, increase resilience, and strengthen food security (IPCC 2023). Complementarily, IPBES emphasizes that the sustainable use of biodiversity, including native and underutilized food species, is essential to secure nature’s contributions to people under global change scenarios (IPBES 2019). These principles are also reflected in FAO agendas, such as the Sustainable Food Systems initiative (Nguyen 2018). In this context, the integration of wild and cultivated plants into diversified production systems emerges not only as an ecological strategy but also as a strategic axis of public policies for the transition toward more resilient, equitable, and sustainable food systems.

Wild food plants (WFPs) are defined as plant species that grow spontaneously in natural or semi-natural ecosystems, have self-sustaining populations (do not require cultivation), and are collected for human consumption (Heywood 1999; Reyes-García et al. 2015; Shackleton and De Vos 2021). They represent important food resources, particularly for local populations. Many WFPs serve as regular components of the diet and income of extractivists and their families or fulfill a complementary role in situations of low agricultural production (Nascimento et al. 2012; Shumsky et al. 2014; Nerfa et al. 2020; Pawera et al. 2020). However, despite the important contribution of WFPs to food diversification (Shumsky et al. 2014; Borelli et al. 2020), it is important to bear in mind that these species may also be threatened by climate change. Studies demonstrated that climate change can reduce suitable areas for WFPs and alter the availability of these species, further aggravating the threat to food and nutrition security (Oliveira et al. 2015; Oluoch et al. 2024; Silva et al. 2024).

Predictive tools such as habitat suitability modeling are important for the identification of climatically suitable and high-risk areas for wild and cultivated species (Peng et al. 2019; Wessels et al. 2021; Hirabayashi et al. 2022; Appelt et al. 2023; Hosseini et al. 2024). A study assessing climatically suitable areas for WFPs in Southern Africa found that declining species richness, combined with reduced agricultural yields, poses a serious risk to the maintenance of food and nutrition security in the region (Wessels et al. 2021). The identification of climatically suitable and high-risk areas for plant development can assist in crop choices and the design of sustainable food systems, which holds particular importance for regions experiencing climate and social vulnerability (Nguyen 2018; Gergel et al. 2020). However, there are no studies that compare WFPs and cultivated food plants (CFPs) in climate change scenarios to understand the role of food diversification as a potential adaptation strategy. Our goal is to fill this gap by using the Brazilian Semiarid region as a model to compare these species groups and assess the potential contribution of WFPs in the future.

The Brazilian Semiarid region faces concerning climate projections, with an average temperature increase of 1–4 °C and a 27% reduction in rainfall by the end of the twenty-first century (Gutiérrez et al. 2023). These conditions can lead to increased water evaporation from reservoirs and lakes, affecting soil moisture and impacting local agriculture, with more prominent effects on rain-dependent CFPs (PBMC 2013; Marengo et al. 2022). In addition to this potential climate vulnerability, the region is home to smallholder farmers who are already facing socioeconomic challenges (Herrera et al. 2018). The Brazilian Semiarid is notable for its high diversity of WFPs, including unconventional species and those of economic importance for extractive activities (Jacob et al. 2020; Silva et al. 2024). In this context, the region serves as a relevant model for comparative analyses between WFPs and CFPs, as well as for investigating the relevance of food diversification as an adaptation strategy to climate change.

This study aimed to examine the potential effect of climate change on habitat suitability for CFPs and WFPs in the Brazilian Semiarid in future scenarios and determine whether food diversification can mitigate the risk of food unavailability (local species loss) in the future. It is expected that there will be a reduction in habitat suitability for both species groups (CFPs and WFPs) in future scenarios, and that this effect will be greater for CFPs. Additionally, the estimated risk is expected to be lower with food diversification, that is, for systems composed of WFPs and CFPs than for systems composed of CFPs only.

## Materials and methods

### Study area

**Fig. 1:**
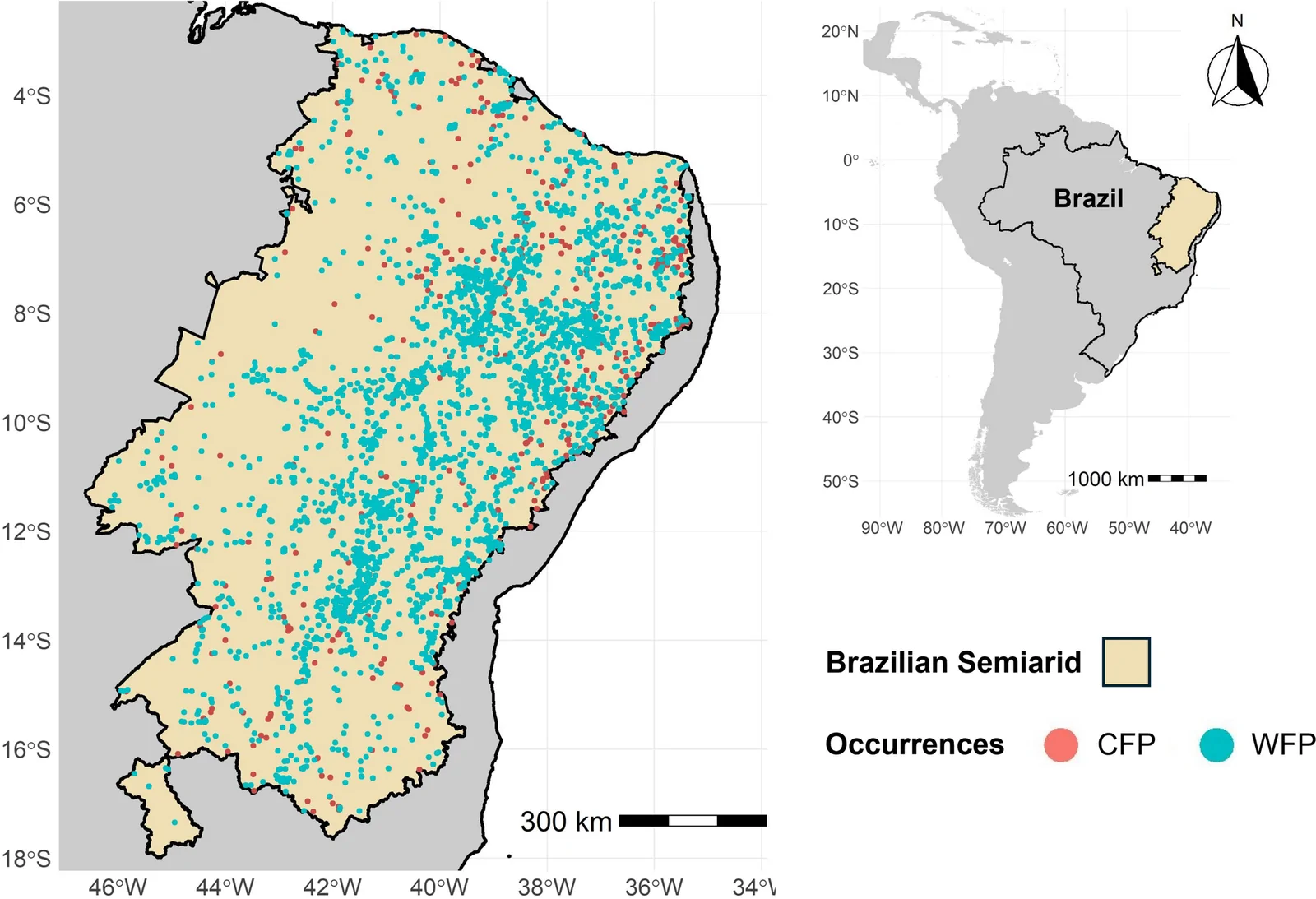
Geographical location of the Brazilian Semiarid within the Neotropical realm. The Brazilian Semiarid is shown with the distribution of the occurrence registers of the wild food plants (WFP) and cultivated food plants (CFP). Sources: digital municipal mesh, territorial areas, and federative units (1:250 000), Brazilian Institute of Geography and Statistics (IBGE 2022); Semiarid borders, Northeast Development Superintendency (SUDENE, 2017); Global Biodiversity Information Facility database (GBIF.org 2022)

The Brazilian Semiarid is characterized by average annual rainfall equal to or less than 800 mm, Thornthwaite acidity index equal to or less than 0.50, and daily percentage of water deficit equal to or greater than 60% (BRASIL 2017). The Brazilian Semiarid accounts for 18% of the national territory and has an estimated population of 27 870 241 people, representing the most densely populated dryland region in the world (Alvalá et al. 2017; SUDENE 2018). It also concentrates the largest number of family farmers in Brazil (IBGE 2017a). Most family farming systems in the region grow rain-dependent crops, such as corn, common bean, and cassava (Melo and Voltolini 2019). The Brazilian Semiarid is home to a rich diversity of WFPs, which are crucial for the economic and dietary needs of local populations, especially extractivists (Lima et al. 2013; Nabout et al. 2016; Jacob et al. 2020) (Fig. 1).

### Habitat suitability modeling

Habitat suitability models provide estimates of the probability of a species occurring under certain environmental conditions based on the relationship between the species' actual occurrences and predictor environmental variables (Guisan et al. 2017). Here, habitat suitability models were constructed according to the method of Silva et al. (2024), with some modifications.

#### Selection and identification of target species

Two groups of food plants with economic and nutritional importance for local populations of the Brazilian Semiarid were selected, one comprising CFPs and the other WFPs. CFPs were selected from the Brazilian Agricultural Census database, available in the automatic recovery system of the Brazilian Institute of Geography and Statistics (IBGE). The following IBGE indexing terms were applied as filters (in Portuguese) within the "Semiarid" region and "family farming" group: "temporary crops," "permanent crops," and "horticulture." All retrieved records (*n* = 129) were subjected to eligibility assessment (IBGE 2017c, 2017d, 2017e). Exclusion criteria were plants not used for human consumption, those used for seedling and seed production, herbs and spices, and leafy vegetables. The exclusion of leafy vegetables was motivated by their considerable dependence on management practices under unfavorable climate conditions, attributed to their shallow roots, limited ability to seek out water, and need for constant watering (Dixon and Aldous 2014; Lopes and Pedroso 2017). Records derived from the same plant, such as cashew fruit and cashew nut, were combined. After eligibility screening, a total of 68 CFPs were selected.

WFPs were also selected from the IBGE database. All plants retrieved by searching the categories "plant extraction," "Semiarid," and "family farming" were selected. This search strategy retrieved plants extracted by family farmers (IBGE 2017b). The IBGE search for WFPs was complemented by using the list of plants reported in the systematic review of Jacob et al. (2020). This complementary sample included all species cited by three or more articles reporting their use in the diet of the Brazilian Semiarid population. After eligibility screening, a total of 11 WFP species were identified in the IBGE database and 17 species in the dataset of Jacob et al. (2020).

As the IBGE database contains only the common names of WFPs and CFPs, the species were identified based on the Flora and Funga of Brazil (Flora e Funga do Brasil 2022). CFPs were also identified according to the National Register of Cultivars of the Ministry of Agriculture, which comprises all cultivars registered for use in Brazil (CultivarWeb 2022). For cases where it was not possible to establish an association between common names and species names, information was sought in the scientific literature and national technical documents. During the identification process, duplicate species and hybrids were excluded, resulting in 62 CFP species and 27 WFP species in this screening step.

#### Records and extent of occurrence of target species

Occurrence records for target species were collected from the Global Biodiversity Information Facility database (GBIF.org 2022). Only records containing complete geographic coordinates and entered from 1970 onward were selected. This initial time limit was used to align with the data collection period of bioclimatic variables. Duplicates were removed. Only records from the Brazilian Semiarid were used for CFPs, assuming that these species are adapted to local conditions through their history of domestication and genetic improvement (Miller and Knouft 2006; Kodis et al. 2018; Lippmann et al. 2019). For WFPs, all records of native occurrence (nationwide) were considered, given that limiting the data to the Semiarid could restrict the sample excessively for modeling purposes (Li and Ding 2016).

It was possible to gain more precise insight into the species cultivated by family farmers by filtering occurrence records by the location (polygons) of small agricultural enterprises. Information on smallholder farms, defined as properties with an area of up to four tax modules, was obtained from the Rural Environmental Register System (SICAR 2022). One fiscal module is a Brazilian territorial extension unit ranging from 5 to 110 ha. Here, it was adopted as a proxy for family farming, as it is one of the official classification criteria for family farms in the country (BRASIL 1979). Filtering of the results yielded a low number of occurrence records for species cultivated in the Semiarid. Therefore, speciesLink (speciesLink 2022) was used as a complementary database. The minimum number of occurrence records for species retention was set at 5 (Velazco et al. 2019). After this process, 26 CFP species were deemed adequate for habitat suitability modeling. The relative contribution of each CFP to the total crop production in the Brazilian Semiarid was calculated. This sample of CFP species accounted for more than half of the agricultural production in the region (~ 63%) and represented about 60% of the species with the highest production volumes (relative contribution > 1%) (Table S1). Occurrence records of WFPs were not filtered by agricultural system, as the aim was to consider their natural distribution in the environment. Thus, the total of food plants comprised of 26 CFPs and 27 WFPs. The nomenclature for CFPs and WFPs follows Plants of the World Online (POWO 2022) and Flora and Funga of Brazil (Flora e Funga do Brasil 2022) (Table S2).

The extent of occurrence (EOO) of each species was determined by drawing minimum convex polygons (MCPs) for occurrence records. For the 27 WFPs, MCPs represent the known native EOO, whereas, for the 26 CFPs, MCPs depict the EOO within the Brazilian Semiarid. A 100 km buffer zone was incorporated to increase the environmental variability for model calibration (Vasquez et al. 2021; Lima et al. 2022).

#### Selection of predictor environmental variables

A total of 19 bioclimatic variables were obtained from WorldClim version 2.0 (Fick and Hijmans 2017; WorldClim 2022c) at a spatial resolution of 2.5 min in the WGS 84 coordinate system for the 1970–2000 period. Data on 11 soil property variables were collected from the SoilGrids database (Poggio et al. 2021; SoilGrids 2022) for the first four depth layers (Wessels et al. 2021) at an aggregate spatial resolution of 5000 m. Soil layers were redesigned and aligned with bioclimatic layers (same projection, resolution, and extension). The initial dataset comprised 60 environmental layers, including 19 bioclimatic variables and 11 soil property variables, with 10 soil variables at four depths (0–5, 5–15, 15–30, and 30–60 cm) and 1 at a single depth (0–30 cm).

Predictor variables were selected using (i) principal component analysis (PCA), (ii) variance inflation factor (VIF), and (iii) Pearson correlation coefficient (*r*). The variables that contributed the most to EOO variance were identified by carrying out separate PCAs for bioclimatic variables (PCA1) and soil property variables (PCA2). The most important variables for the formation of PCA1 and PCA2 axes were then identified and selected (Kassambara 2020; Kassambara and Mundt 2020). Only variables with a VIF ≤ 3 were retained to minimize collinearity among those selected from the two PCAs (Zuur et al. 2010; Dormann et al. 2013). Finally, a correlation test was performed using the occurrence values of each species, and variables with an *r* ≤ 0.7 were selected. Table S3 provides a detailed description of the bioclimatic and edaphic variables included in the final models for each species.

Projections for future climate change scenarios were made using the same bioclimatic variables used for the construction of each model. This analysis was conducted based on global climate models available in WorldClim (WorldClim 2022b) at a spatial resolution of 2.5 min, for the 2041–2060 period. The same soil layers described above were used for future projections.

#### Model development and prediction

Habitat suitability models were constructed using a maximum entropy algorithm (MaxEnt) (Phillips et al. 2006; Elith et al. 2011). MaxEnt is a machine learning algorithm frequently used in this type of analysis for its satisfactory performance compared with other algorithms (Valavi et al. 2022). Models were adjusted using the following parameters: Jackknife (5 to 25 occurrence records) and block (> 25 occurrence records) methods, 500 maximum iterations, 10 000 background points (randomized in the EOO of each species), 10 values (ranging from 0.5 to 5 with increments of 0.5) of regularization multiplier (RM), and six feature classes (FC) with increasing complexity (Merow et al. 2013; Muscarella et al. 2014). The different combinations of FC and RM generated a total of 60 models for each species. Model selection was based on the lowest value of the corrected Akaike information criterion (AICc) combined with acceptable values of the area under the receiver operating characteristic curve (AUC > 0.7) (Elith et al. 2011; Warren and Seifert 2011; Muscarella et al. 2014). Thus, a single adjusted model was obtained for each species (Table S3).

Climate projections from 11 global climate models (GCMs) were used (Table S2). These computational models, developed by several research centers, simulate climate in different layers of the atmosphere (WorldClim 2022a). Furthermore, four shared socioeconomic pathways (SSPs) were proposed, namely SSP1-2.6, SSP2-4.5, SSP3-7.0, and SSP5-8.5. These climate change scenarios are based on different levels of greenhouse gas emissions and socioeconomic assumptions, including population, technological, and economic growth (Hausfather 2019). Models were designed for each species in all future climate models and scenarios (11 GCMs × 4 SSPs), and one model was chosen by taking into account the mean of GCMs for each scenario (4 SSPs). This approach was adopted to encompass uncertainties related to future climate conditions. The spatial projections of these models were sectioned according to the Brazilian Semiarid, resulting in five maps of habitat suitability surfaces for each species, one for the current scenario and four for future scenarios. The absolute variation of habitat suitability was calculated as the difference between climatic scenarios (Future−Current) for each SSP and species.

### Estimation of species number

Continuous habitat suitability data were converted to a binary distribution using the maximum training sensitivity plus specificity (MTSS) threshold. Pixels with values above the threshold were considered to indicate presence (1) of the species and those with values below the threshold were considered to indicate absence (0) of the species (Table S3, Fig. S1). The MTSS threshold is recommended for its good performance in identifying suitable areas (Liu et al. 2016). Binarization was performed for each species (individually, as each one has a specific threshold) and for all climate scenarios. For determination of the number of species, binary maps were superimposed, and the number of species in each pixel was added for each climate scenario (current and future). The relative variation of species number between climate scenarios was calculated as follows: Future−Current/Current × 100. This variable was estimated for CFPs, WFPs, and cultivated and wild food plants (CWFPs).

### Data analysis

The impact of future climate change on food plants occurring in the Brazilian Semiarid was tested by determining whether the absolute variation of habitat suitability (difference between future and current scenarios) was different from zero. For this, the one-sample Student's *t* test was used for all species in each future scenario. Positive values indicate an increase in habitat suitability, and negative values indicate a reduction. Identification of the group of species most negatively affected by climate change was achieved by comparing the absolute variation of habitat suitability between CFPs and WFPs in each future scenario, using a generalized linear mixed model. For the response variable, a random and paired sample of the absolute variation of suitability values of 1000 pixels was extracted for each species (26 cultivated and 27 wild), totaling 53 000 replicates (26 000 for cultivated species and 27 000 for wild species) for each future scenario. Thus, species group (CFP and WFP) was used as a predictor variable (fixed effect), and pixel identity was included as a random effect to account for the spatial variability. This analysis was performed for each of the four future scenarios.

The variation in the projected risk of CFP, WFP, and CWFP unavailability was determined to assess whether food diversification can reduce species unavailability. The negative relative variation (− 1% to − 100%) of the number of species in each group was used as the species unavailability risk proxy for each future scenario. The Kruskal–Wallis test (Wilcoxon post hoc test) was performed using the projected climate risk (loss of species) as the response variable and species group as a predictor variable for each future scenario. For determination of the response variable, random and independent samples of the negative relative variation of species number in 1000 pixels were taken for each species group (CFP, WFP, and CWFP), totaling 3000 replicates (1000 pixels per group) for each future scenario.

QGIS software version 3.16.16 (Qgis Development Team 2021) was used for the processing and visualization of geographic information. The R environment version 4.1.2 (R Core Team 2021) was used for habitat suitability models, mixed linear models, other statistics, and the generation of maps and graphs. For the selection of predictor variables, PCA was performed using the FactoMineR (Lê et al. 2008) and factoextra (Kassambara and Mundt 2020) packages. Parameter selection for habitat suitability models was carried out using the ENMeval package (Kass et al. 2021). The MaxEnt algorithm was implemented using the dismo (Hijmans et al. 2023) and maxnet (Phillips 2021) packages. For mixed linear models, the lmerTest package (Kuznetsova et al. 2017) was applied. The *t* test and Kruskal–Wallis test were performed using the rstatix (Kassambara 2020) and PMCMRplus (Pohlert 2023) packages.

## Results

### Variation in habitat suitability

**Fig. 2:**
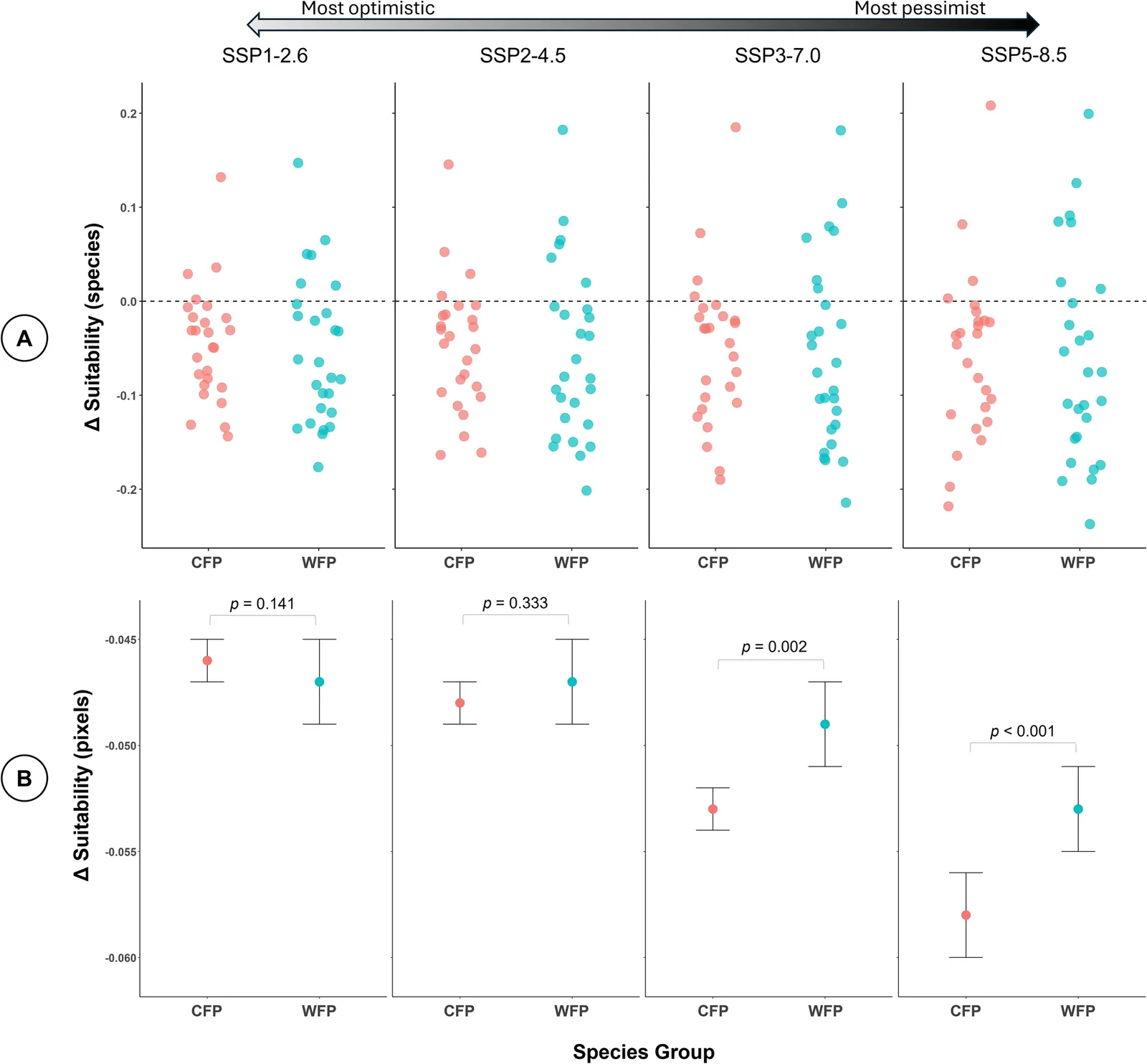
**A** Variation in habitat suitability for cultivated (CFPs, *n* = 26) and wild (WFPs, *n* = 27) food plants in future scenarios (SSPs) of climate change (2041–2060) in the Brazilian Semiarid. Positive values indicate increased habitat suitability, and negative values indicate reduced habitat suitability. **B** Means and confidence intervals of the variation in habitat suitability pixels (*n* = 1000) per species group in each future scenario

In general, the predictions indicated a significant negative variation in habitat suitability within the Brazilian Semiarid under future climate scenarios, suggesting a reduction in suitable habitats for the analyzed food plants (*n* = 53), rather than an expansion (Fig. S2, Table S4). About 79% of species (*n* = 42) showed a reduction in the mean pixel values of habitat suitability in all future scenarios; of these, 9 exhibited reductions above 50% in the most pessimistic scenario (SSP5-8.5) (Table S5). In stratifying by species group (26 CFPs and 27 WFPs), it was observed that 85% of CFPs (*n* = 22) and 74% of WFPs (*n* = 20) showed a reduction in mean habitat suitability in all future scenarios (Fig. 2A, Table S5). Although there was a reduction in suitability in both groups, WFPs had a lower negative variation than CFPs in the most pessimistic scenarios of greenhouse gas emissions, namely SSP3-7.0 (Linear Mixed Model: *t* = 3.14, *p* = 0.002) and SSP5-8.5 (Linear Mixed Model: *t* = 3.95, *p* < 0.001) (Fig. 2B, Table S6).

**Fig. 3:**
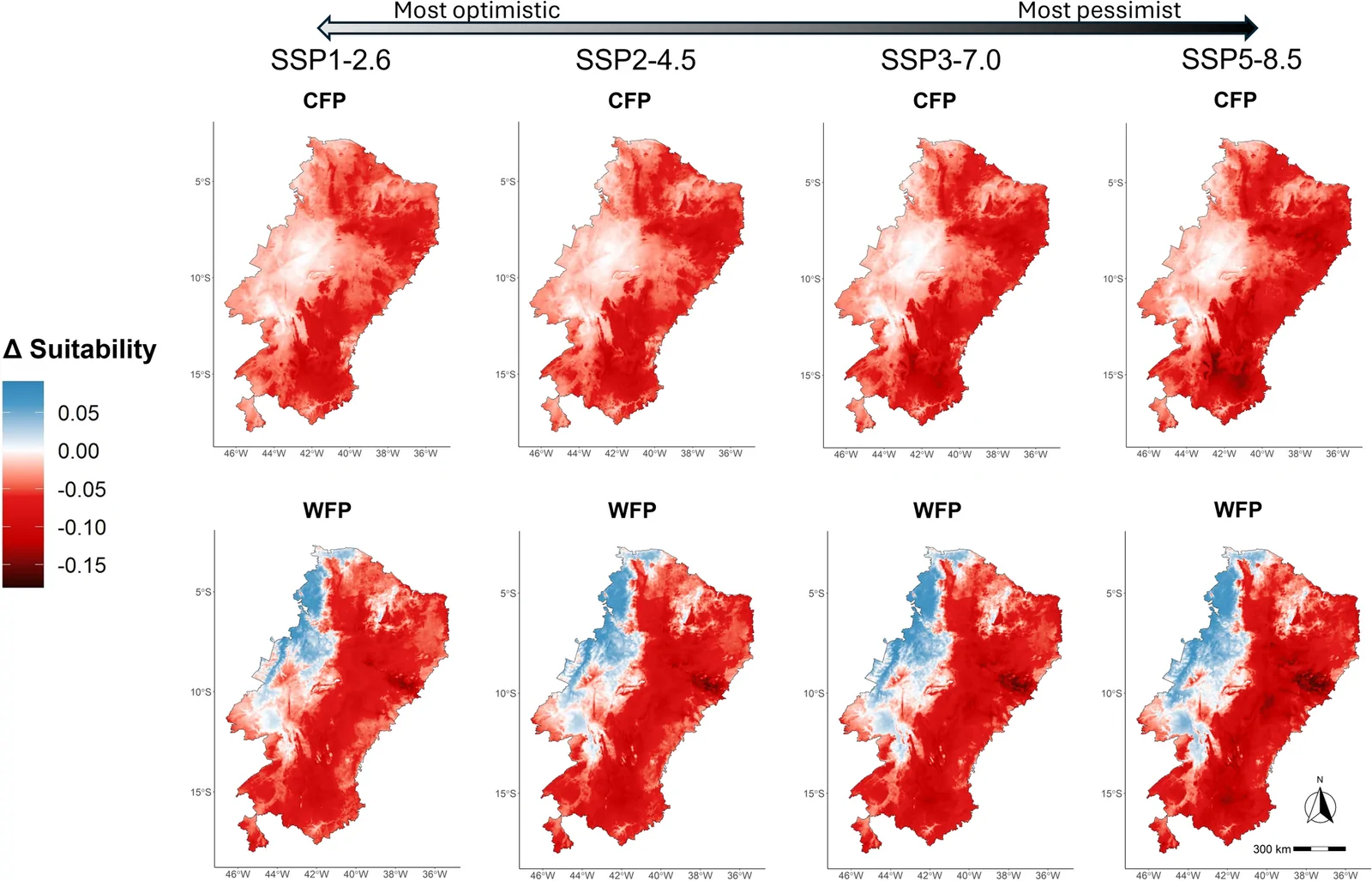
Spatial projection of the variation in habitat suitability for cultivated (CFPs) and wild (WFPs) food plants in future scenarios (SSPs) of climate change (2041–2060) in the Brazilian Semiarid. The variation in habitat suitability pixels refers to the mean variation for species composing each group (CFP, *n* = 26; WFP, *n* = 27) in each future scenario. Positive values indicate increased habitat suitability, and negative values indicate reduced habitat suitability

The variation in habitat suitability for food plants in the Brazilian Semiarid was similar among future climate scenarios. However, habitat suitability differed spatially between CFPs and WFPs. Areas with negative variation were distributed throughout almost the entire Semiarid, but the eastern part had the greatest reductions in habitat suitability for both groups (Fig. 3). The western area, by contrast, exhibited the lowest variations in habitat suitability for both groups (Fig. 3). In this region, variations were close to zero, but WFPs had positive values.

### Potential risk of species unavailability

**Fig. 4:**
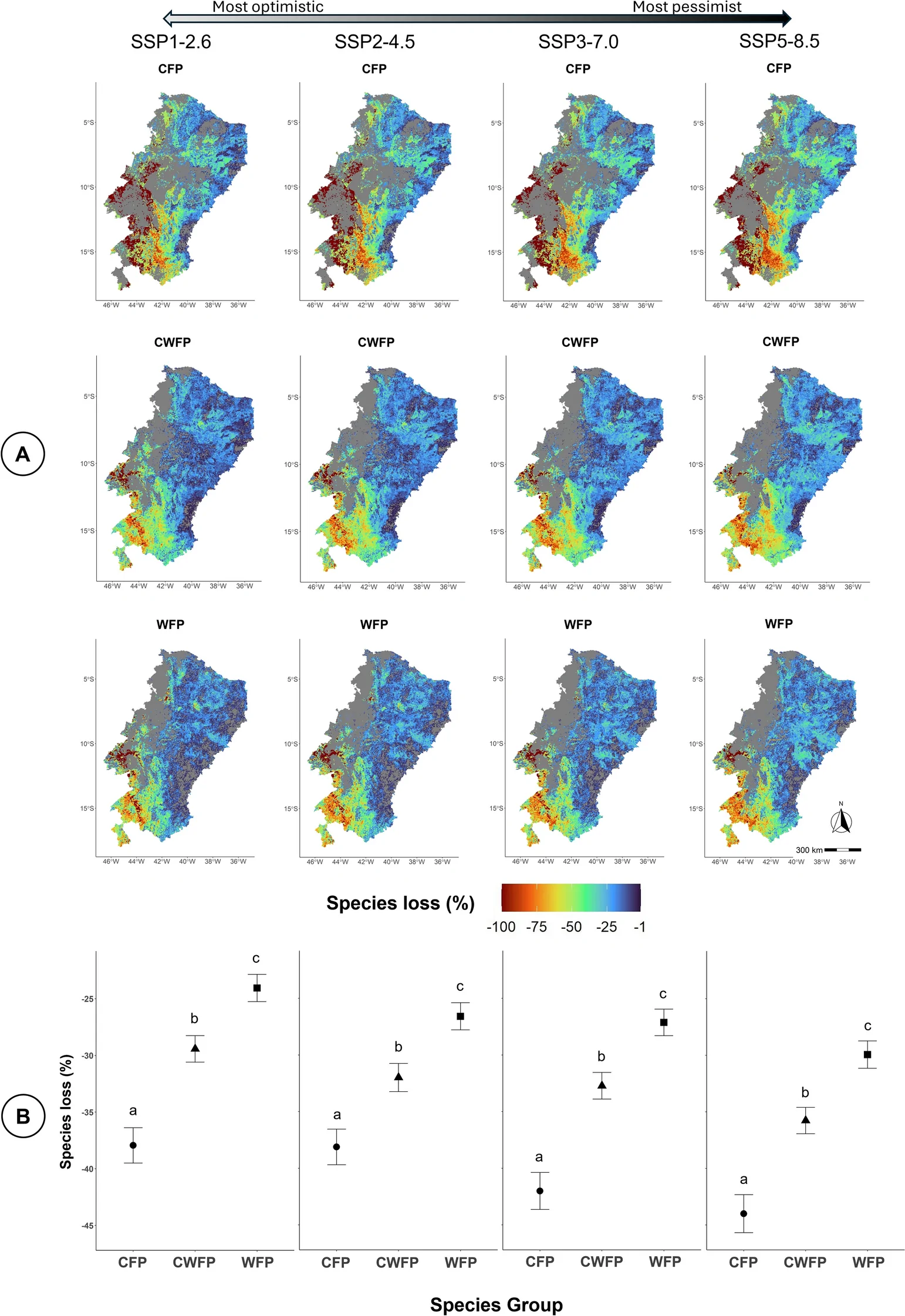
Risk of species unavailability for cultivated (CFPs), wild (WFPs), and cultivated and wild (CWFPs) food plants in four future scenarios (SSPs) of climate change (2041–2060). **A** Projected risk of species unavailability in the Brazilian Semiarid. **B** Means and confidence intervals of the risks of species unavailability. The risk of species unavailability is represented by the negative relative variation (from − 1 to − 100%) of the number of species (species loss) in each pixel (*n* = 1000). Different lowercase letters indicate statistical differences between groups according to the Wilcoxon post hoc test (*p* < 0.001)

The risks of species unavailability were projected over almost the entire Semiarid in the four future scenarios for all species groups (Fig. 4A). Spatially, the highest projected risks (species loss > 50%) were mainly located in the midwest and southwest regions of the Semiarid, whereas the lowest risks were observed in the eastern part. The highest risk areas coincide with the areas with the lowest numbers of species (midwest and southwest) for both groups (Fig. S3). The magnitude of risks differed between groups, with CFPs having the greatest species loss in all future scenarios compared with WFPs and CWFPs: SSP1-2.6 (Kruskal–Wallis: *X*^2^_(2)_ = 239.3, *p* < 0.001), SSP2-4.5 (Kruskal–Wallis: *X*^2^_(2)_ = 146.4, *p* < 0.001), SSP3-7.0 (Kruskal–Wallis: *X*^2^_(2)_ = 218.7, *p* < 0.001), and SSP5-8.5 (Kruskal–Wallis: *X*^2^_(2)_ = 179.1, *p* < 0.001) (Fig. 4B).

## Discussion

The results indicate that the climate changes projected for 2041–2060 may negatively impact the availability of food plants, both cultivated and wild. Although this study used the Brazilian Semiarid as a model region, the results are consistent with global trends, suggesting that similar effects may occur in other dry regions of the world. Recent studies have also documented future reductions in climatically suitable areas for food plants in several countries (Hirabayashi et al. 2022; Appelt et al. 2023; Oluoch et al. 2024). The reduction in suitability may affect not only ecosystems but also the livelihoods of local human populations that depend on these resources, especially extractivists and smallholder farmers (Evangelista-Vale et al. 2021; Wessels et al. 2021). Thus, future reductions in food plant suitability may compromise food security and increase socioeconomic vulnerability, pointing to the need for more effective adaptation strategies.

Projections indicated that cultivated food plants (CFPs) may be more negatively affected than wild food plants (WFPs). This result can be explained by the fact that many modern cultivated species exhibit adaptations to more restricted environmental conditions (Khoury et al. 2022; Gao et al. 2023). Species with smaller geographic distributions experience lower climatic variability and are therefore more vulnerable to climate change (Ohlemüller et al. 2008). Cultivation sites also tend to have low soil water retention and high susceptibility to aridity, conditions that are expected to worsen for rainfed crops in the Semiarid (Steward et al. 2018; Rattis et al. 2021). In addition, rising temperatures can impose strong negative effects by disrupting key physiological and reproductive processes, such as pollen viability, fertilization, and grain or fruit filling, even under conditions where water is not limiting (Bita and Gerats 2013; Hatfield and Prueger 2015). The projected reduction in suitability suggests that, in the future, cultivation of these species may require greater use of agricultural inputs, heat-tolerant cultivars, or increased irrigation, as already projected for the hottest regions of the planet (Zhu et al. 2022).

Although our projections do not include physiological responses to rising atmospheric CO_2_, some studies show that crops such as cassava, yam, and some legumes exhibit increased photosynthetic efficiency, water-use efficiency, and biomass accumulation under elevated CO_2_ concentrations (Leakey et al. 2009; Wessels et al. 2021). These responses may partially offset climate-related declines for some species, although the magnitude and consistency of these effects vary widely among taxa and environmental conditions (Bernauer et al. 2024). However, the lack of spatially explicit data on species’ responses to CO_2_ that would allow robust integration of this effect made it impossible to incorporate these mechanistic processes into the modeling. Therefore, our projections should be interpreted as estimates of future habitat suitability for the availability of the analyzed species, focusing on climate-driven constraints rather than as direct predictions of the productive performance of these species.

The reduction in CFP suitability may directly affect the survival and quality of life of smallholder farmers, given their strong dependence on agriculture for subsistence and income (Habtemariam et al. 2017; Rama et al. 2022). However, part of these impacts may be mitigated by the adoption of heat- and drought-tolerant cultivars, including traditional local varieties, which exhibit high genetic diversity and climatic resilience (Perales et al. 2003; Zimmerer 2010). Because these adaptive options and ecophysiological responses were not included in the models, CFP vulnerability is likely to have been partially overestimated, and our results should be interpreted as representing the current situation in the absence of adaptive interventions. Even so, access to and adoption of resilient cultivars depend on socioeconomic conditions, local seed systems, and technical assistance, which vary widely among rural communities.

In some cases, income reductions associated with CFP declines may be offset by opportunities related to WFPs. For example, although economically important cultivated species in the Semiarid, such as *Passiflora edulis* Sims (passion fruit, fruit) and *Anacardium occidentale* L. (cashew, nut), may experience future suitability losses, wild species from similar food groups, such as *Passiflora foetida* L. (wild passion fruit, fruit) and *Dipteryx alata* Vogel (baru, nut), may maintain or expand their suitability, preserving up to 80% of their current area (Silva et al. 2024). However, it is necessary to promote farmers’ interest and consumer demand for WFPs, given the challenges in accepting these plants as food sources (Souza et al. 2023). One approach to this issue is the implementation of programs to popularize WFPs. It is also relevant to investigate the nutritional value of WFPs in order to assess their potential to ensure food and nutritional security under climate change.

Although WFPs generally show greater climatic resilience, our results indicate that several of these species will also suffer significant reductions in suitability, which may increase risks of overexploitation, population collapse, and local loss if collection demand grows in response to CFP fragility. The sustainability of extractive activities depends on the integration of different knowledge sources, as well as the implementation of public policies suited to local contexts (Fromentin et al. 2023). In the Brazilian Semiarid, for example, researchers show that extraction of mangaba (*Hancornia speciosa* Gomes), traditionally carried out by smallholder farmers, can remain sustainable if harvesting does not exceed 87% of the available fruits (Lima et al. 2013). Therefore, public policies and sustainable management strategies should include harvest limits, participatory monitoring, and strengthening of value chains for these species (Talucder et al. 2024), as well as their integration into biodiverse agroecosystems that promote ecological restoration (Santos et al. 2019).

The results showed that the projected high-risk areas for CFPs were larger when compared to the areas that consider both groups combined (crop and wild food plants). The diversification of food species and productive landscapes can increase the availability of food and nutrients (Powell et al. 2015; Gergel et al. 2020). Thus, increasing the diversity of food systems can contribute to climate change adaptation, since food availability and stability are central dimensions of food and nutritional security (FAO 2008). Future research should also evaluate the remaining dimensions of food security, access and utilization, to provide a more comprehensive assessment beyond availability. Agroecological strategies such as agroforestry, crop rotations, intercropping, and conservation agriculture can incorporate both WFPs and CFPs, increasing ecological resilience, soil fertility, and microclimatic stability (Altieri and Nicholls 2017; FAO 2021; Su et al. 2021). In addition, some CFPs showed stability or even gains in suitability in certain subregions of the Semiarid, indicating that WFPs should be understood not as substitutes but as co-benefits that strengthen sustainable agricultural systems, especially where crop composition is predominantly vulnerable.

More broadly, the results of this study directly dialog with international political and scientific frameworks that recognize the diversification of food systems as a central strategy to address the interconnected crises of climate change, biodiversity loss, and food insecurity. Recent IPCC and IPBES reports emphasize that food systems based on local biodiversity, including the valorization of native, wild, and underutilized species, increase socioecological resilience, reduce vulnerabilities, and promote multiple environmental, nutritional, and socioeconomic co-benefits (IPBES 2019; IPCC 2023). In this sense, our results support these international guidelines by providing spatially explicit evidence that the integration of cultivated and wild food plants can reduce climate risks and increase the stability of food systems in vulnerable regions. By quantifying potential suitability gains and reductions in high-risk areas through diversification, this study contributes to supporting public policies aimed at ecosystem-based adaptation, the conservation of agrobiodiversity, and the strengthening of rural livelihoods. Although the impacts of these strategies are strongly dependent on local ecological, institutional, and cultural realities, their adoption across different territories can generate relevant cumulative effects, contributing to the simultaneous advancement of climate adaptation, biodiversity conservation, and food security goals.

Although widely used and recognized as robust tools for spatial analyses, habitat suitability models have limitations, especially when applied to cultivated plants. The relatively small number of occurrence records for some species may restrict the representation of their environmental variability, increasing uncertainty in estimates (van Proosdij et al. 2016). To minimize this bias, we adopted the minimum number recommended for regional analyses (Velazco et al. 2019) and used the Brazilian Semiarid as the background area of the models (Miller and Knouft 2006), which reduces geographic distortions but may still limit the representation of intraspecific genetic diversity and the potential adoption of more resilient cultivars. In addition, the models do not incorporate physiological responses to rising CO_2_ or the effects of adaptive agricultural practices, so the projections should be interpreted as conservative estimates of exposure to climate risk rather than direct predictions of productivity. This choice is methodologically consistent with the objective of mapping the intrinsic climatic sensitivity of species at the regional scale. Moreover, it avoids overly optimistic biases associated with the implicit assumption of uniform technological adaptation, especially in smallholder agriculture contexts where access to improved seeds, irrigation, and inputs is highly unequal. The projected absences in the models should therefore be treated as pseudo-absences, since technological advances, genetic improvement, or management changes may enable cultivation in areas currently considered unsuitable. In this context, the adoption of a medium-term time horizon and conservative scenarios provides a more spatially explicit and useful basis to guide public policies, adaptation strategies, and the prioritization of actions in vulnerable regions.

## Conclusion

The climate changes predicted for the next 30 years (2050) may negatively impact both CFPs and WFPs. The greatest negative effects are expected in areas that are climatically suitable for CFPs rather than WFPs. Areas with greater risks of species unavailability were reduced when CFPs were associated with WFPs. From this perspective, the diversification of food systems is crucial for conserving biodiversity and ensuring food security in the future. The results underscore the importance of restoring and conserving native forests to sustain the future viability of extractive activities involving WFPs. Additionally, there is a need for public policies that support sustainable extractivism and promote the use of WFPs. We emphasize that expanding research to other vulnerable regions, mapping habitat suitability, and identifying species with nutritional potential can contribute to global strategies that increase adaptation and mitigate the effects of climate change. Such efforts should also integrate multidimensional assessments of food security, including access to and utilization of food, in order to strengthen the evidence base for effective and equitable adaptation planning.

## Supplementary Information

Below is the link to the electronic supplementary material.

Supplementary file1 (PDF 3992 KB)

## Data Availability

All occurrences species data used for this study are open access and available in: https://www.gbif.org/occurrence/search?occurrence_status=present and https://specieslink.net/search/. All environmental layers are also open access and available in: https://www.worldclim.org/data/index.html and https://files.isric.org/soilgrids/latest/data_aggregated/5000m/.
