# Supplementary material for: Food diversification as an adaptation strategy to climate change: Habitat suitability for wild and cultivated food plants in the Brazilian Semiarid
Source: Ambio. 2026 Apr 4;55(10):2451–65. doi: 10.1007/s13280-026-02374-2 (PMC13554029; doi:10.1007/s13280-026-02374-2)
Supplement: Supplementary file 1 [file 13280_2026_2374_MOESM1_ESM.pdf]

AMBIO

Food diversification as an adaptation strategy to climate change: habitat suitability for wild and cultivated food plants in the Brazilian Semiarid

Electronic Supplementary Material: Figures and Tables

Figure S1. Maps of binarized habitat suitability models (presence, absence), in the current climate scenario (1970-2000) with species occurrence records in the Brazilian Semiarid. Occurrence records (black dots) of the 27 wild food plants and 26 cultivated food plants were obtained from the Global Biodiversity Information Facility - GBIF (GBIF, 2022).

Wild Food Plants

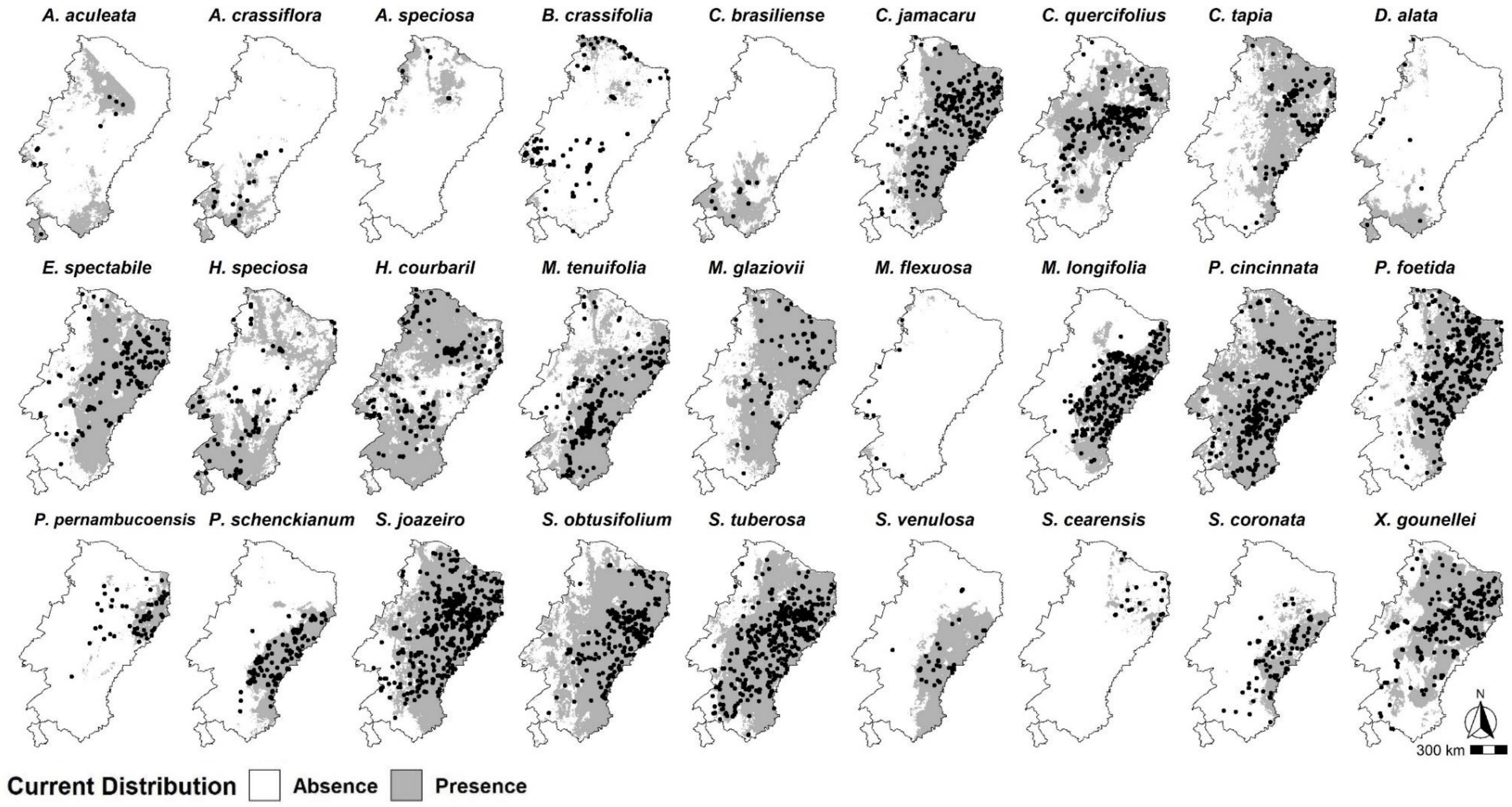

Cultivated Food Plants

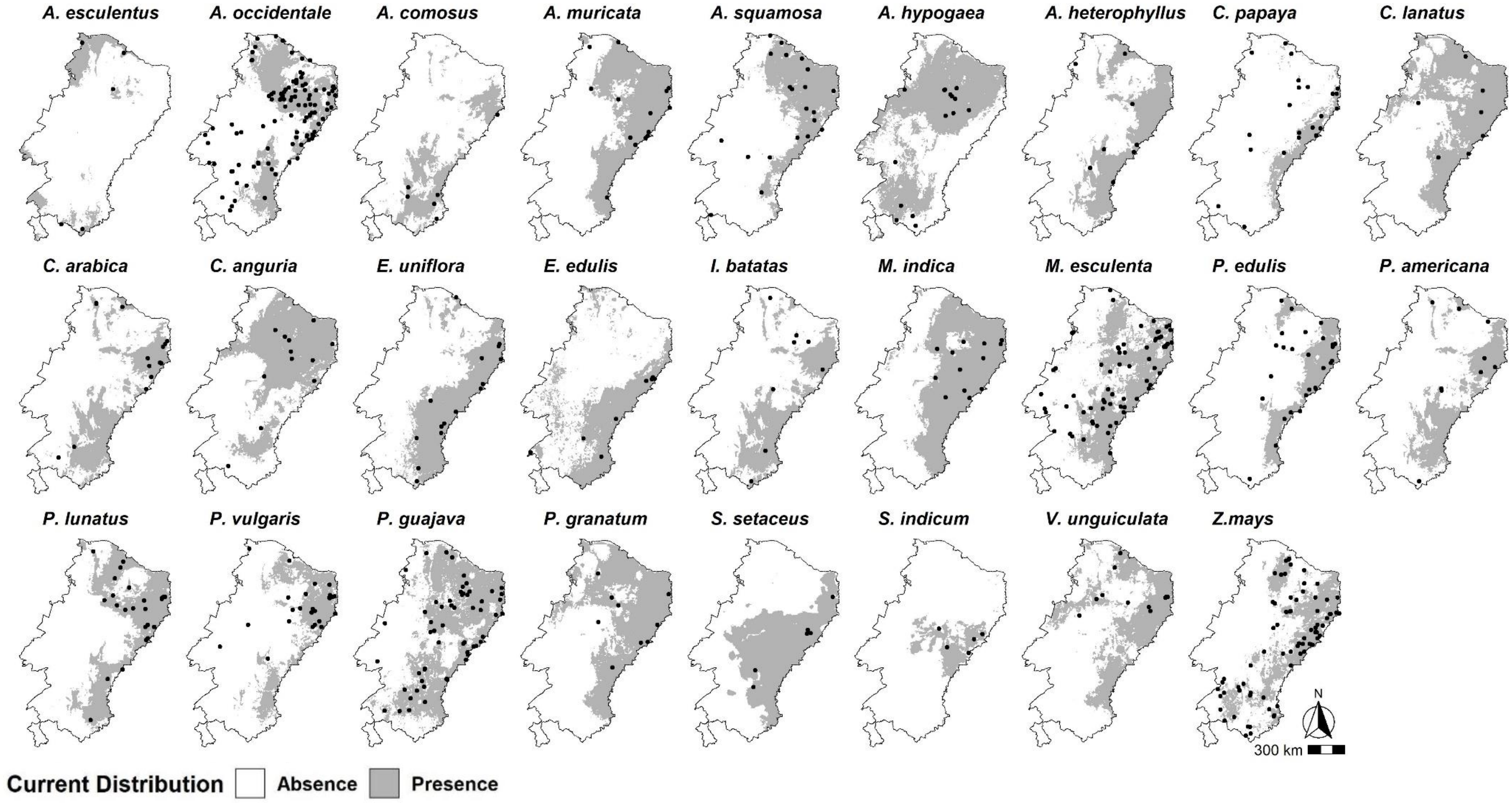

Figure S2. Variation in habitat suitability for cultivated and wild species in the Brazilian Semiarid in the four climate change scenarios (SSPs) (2041-2060). The variation was calculated by the difference between climate scenarios (future - current). Positive values indicate an increase and negative values indicate a reduction in habitat suitability for the species.

SSP1-2.6 – Wild Food Plants

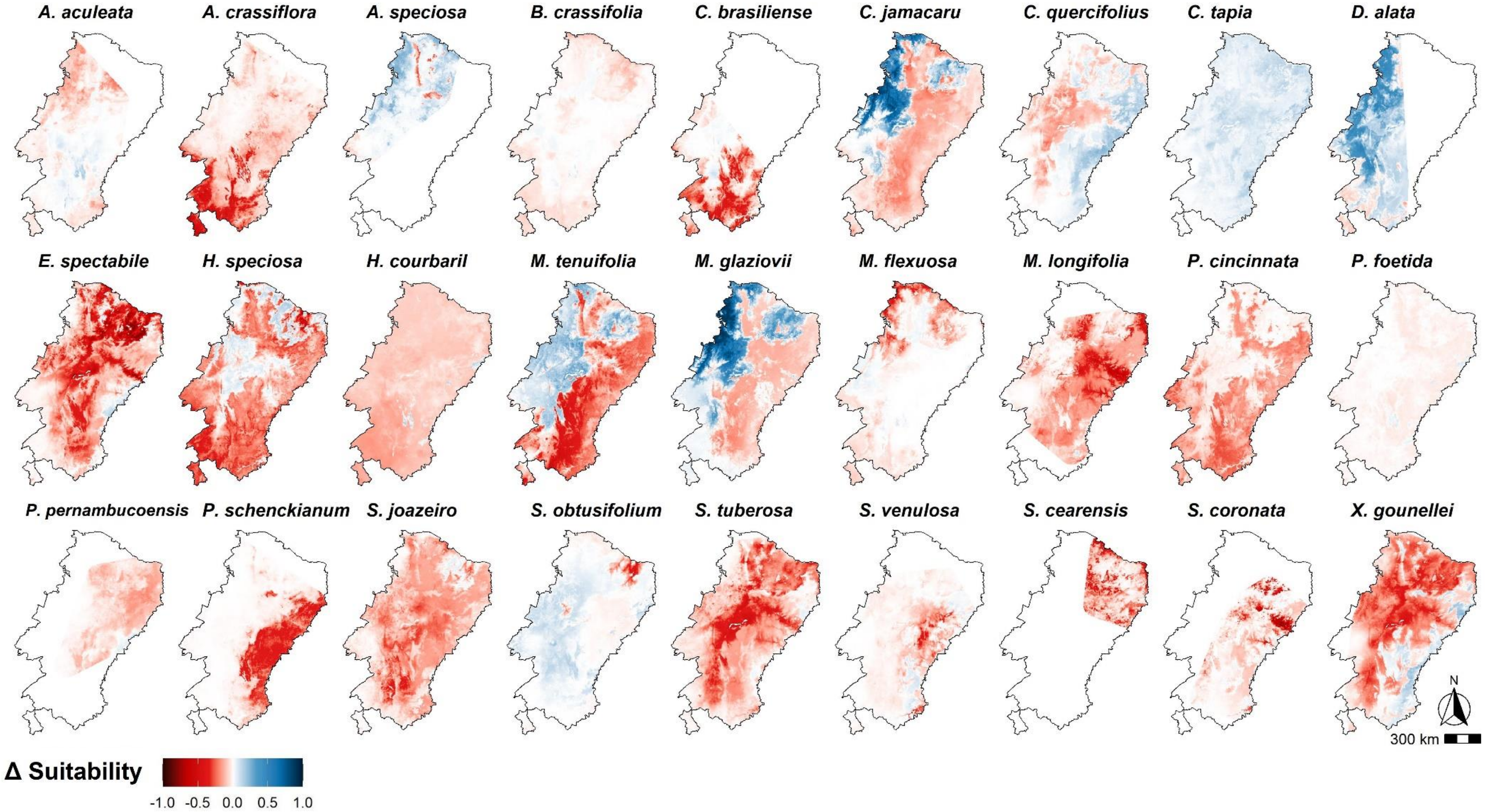

SSP1-2.6 – Cultivated Food Plants

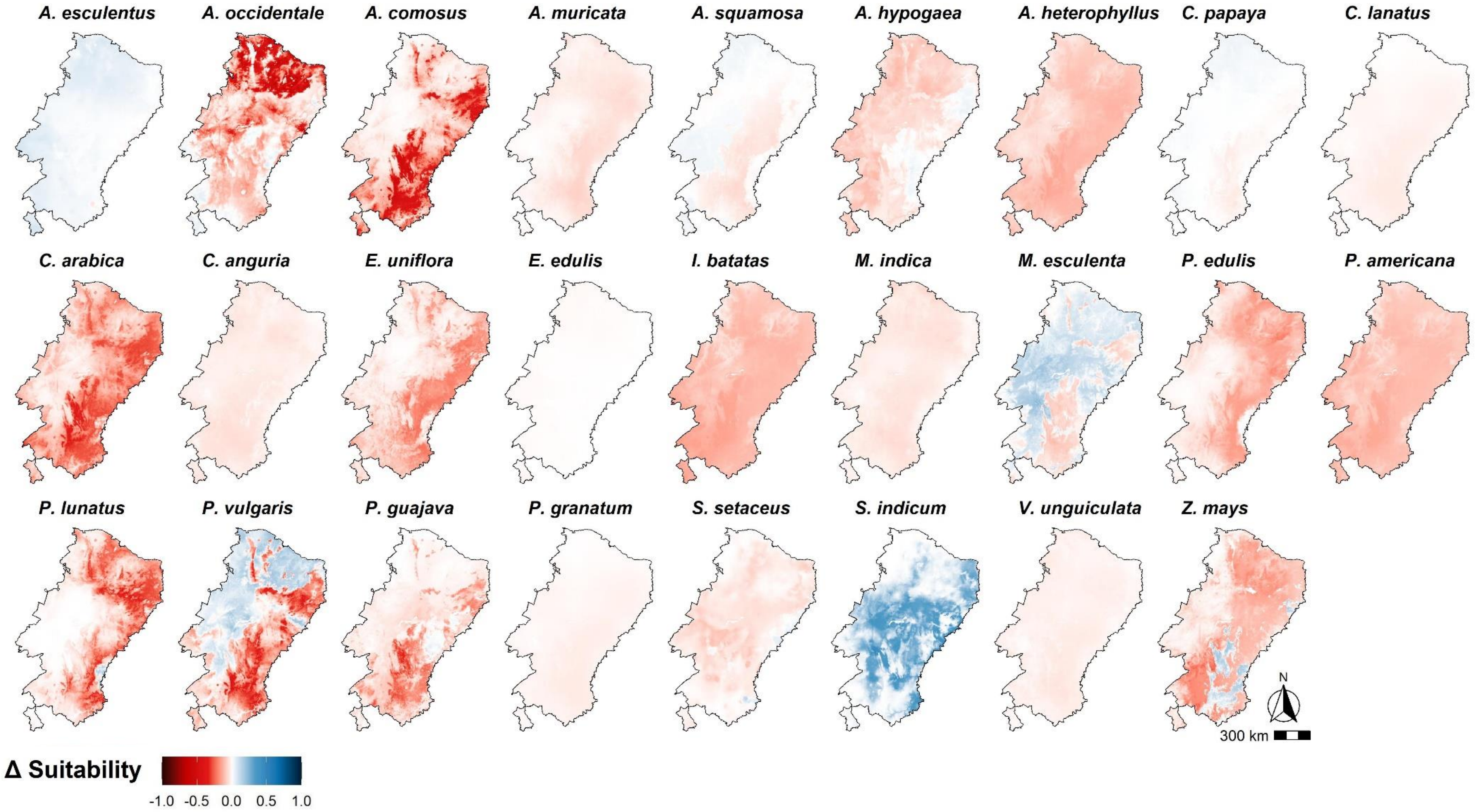

SSP2-4.5 – Wild Food Plants

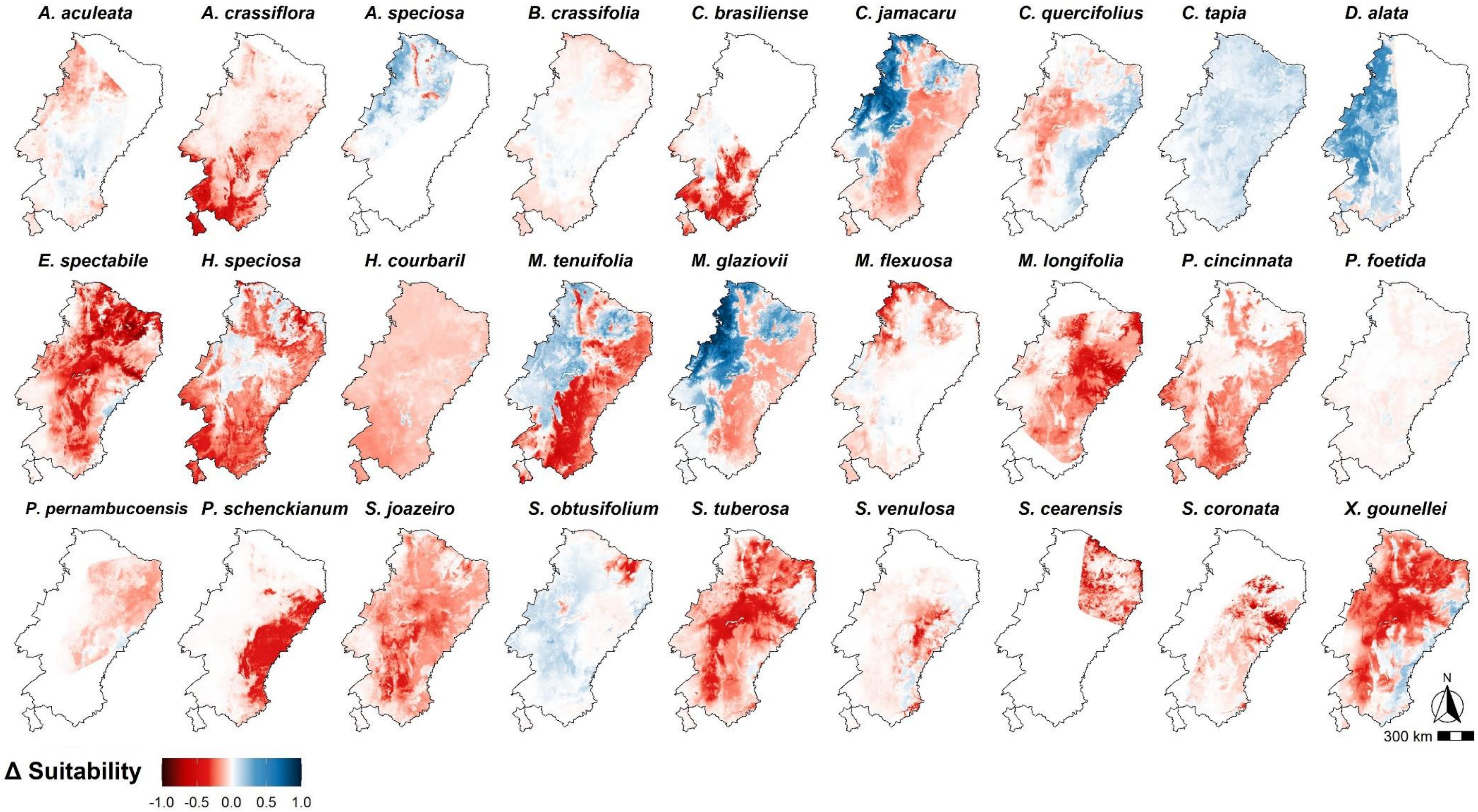

SSP2-4.5 – Cultivated Food Plants

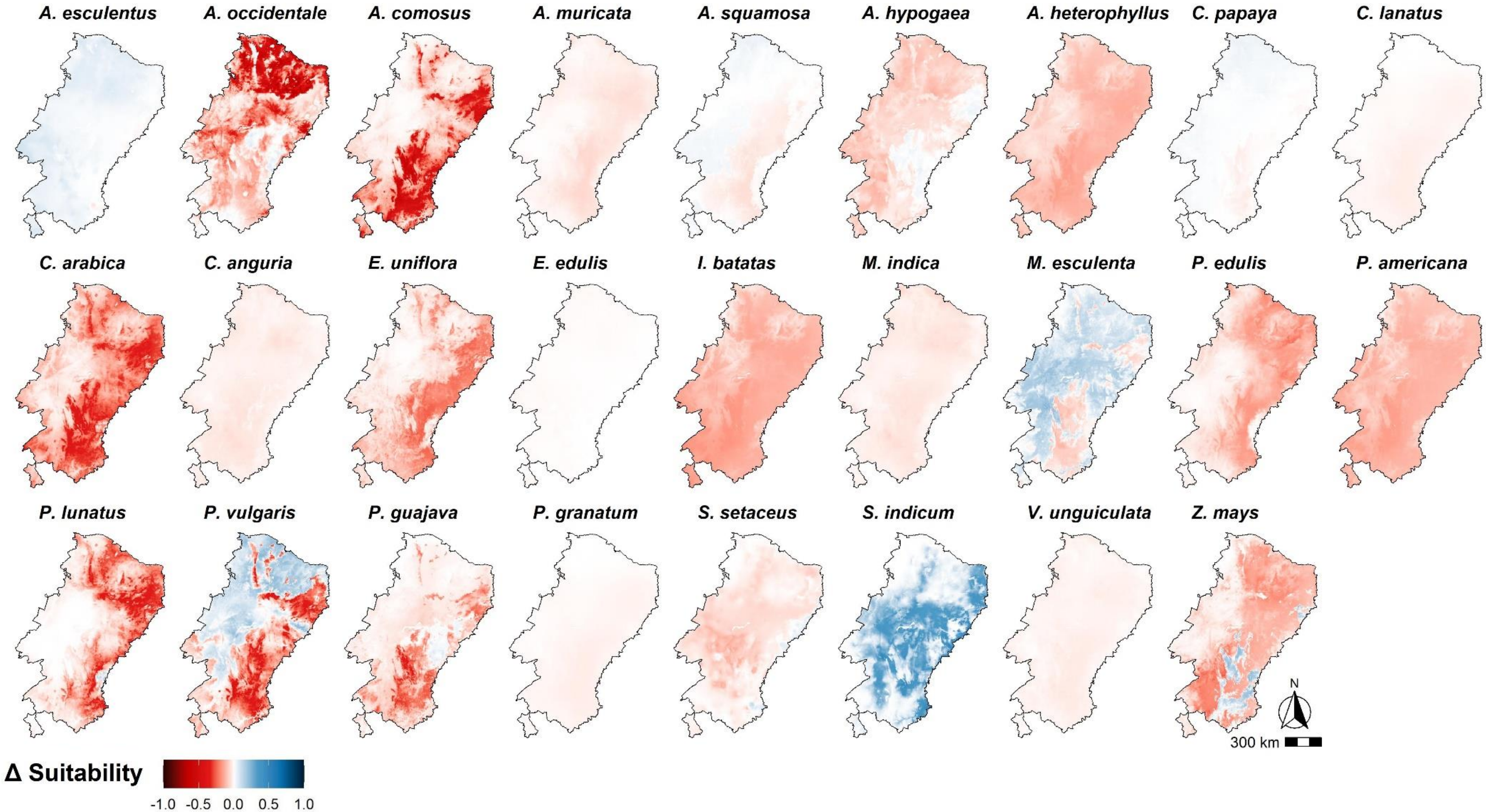

SSP3-7.0 – Wild Food Plants

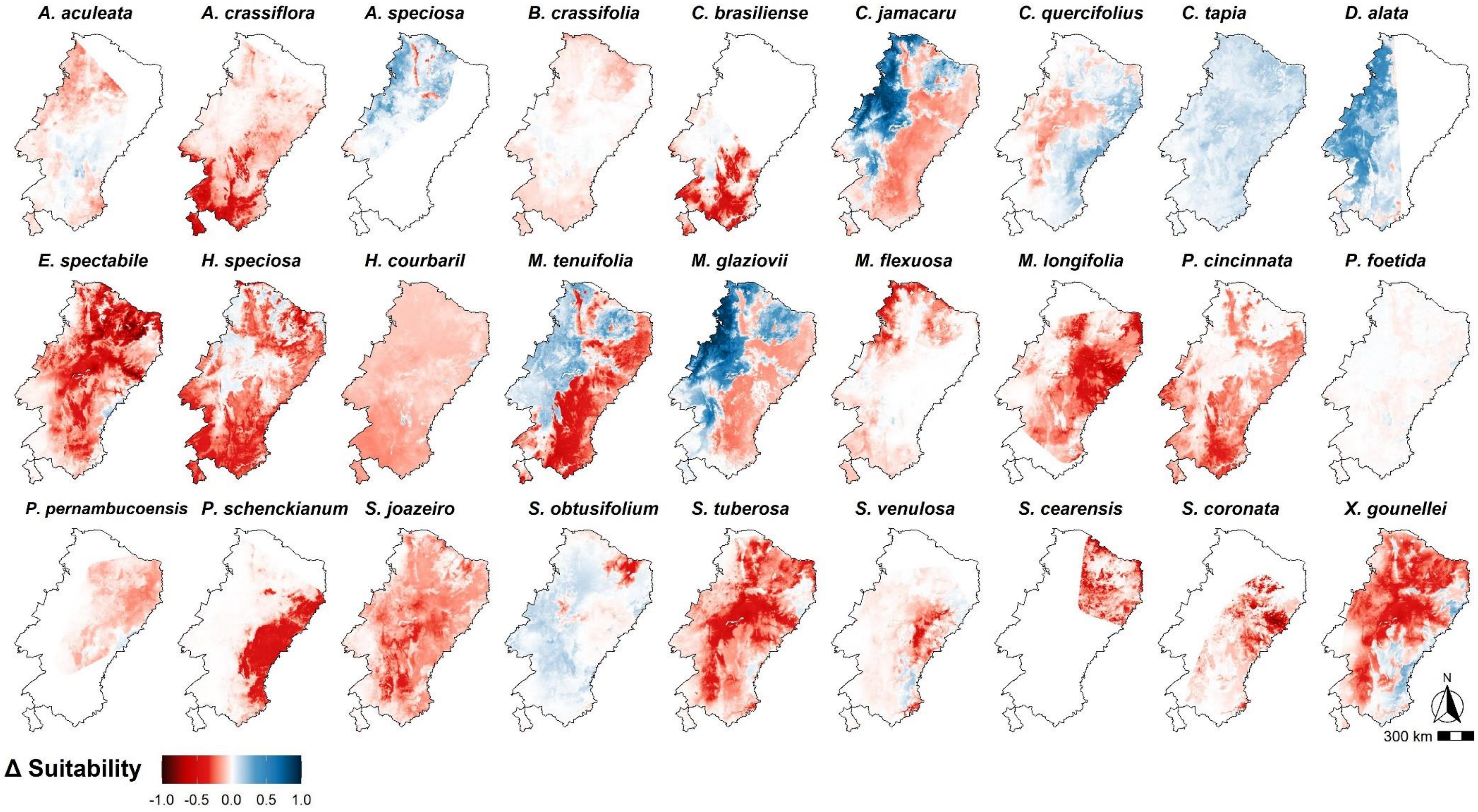

SSP3-7.0 – Cultivated Food Plants

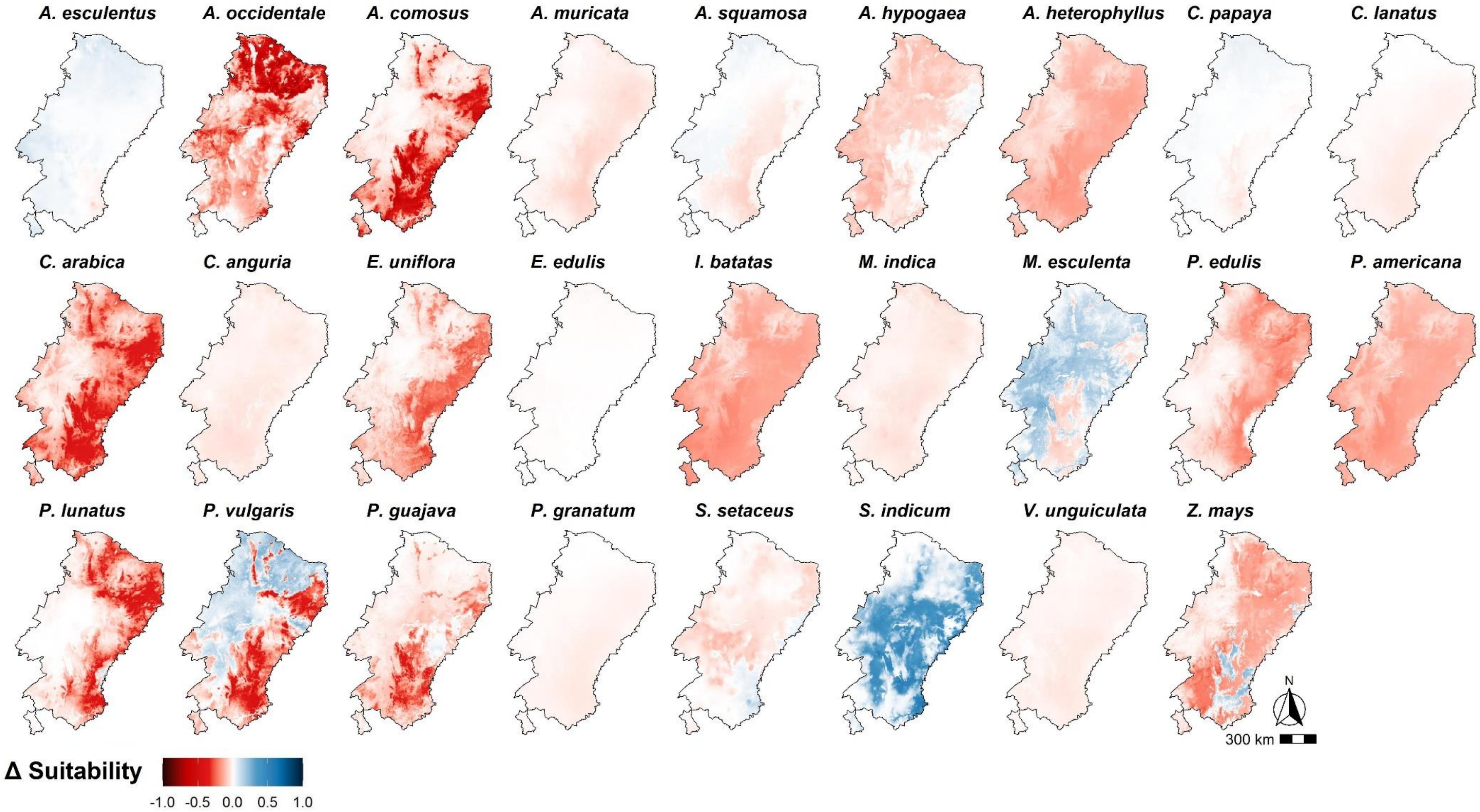

SSP5-8.5 – Wild Food Plants

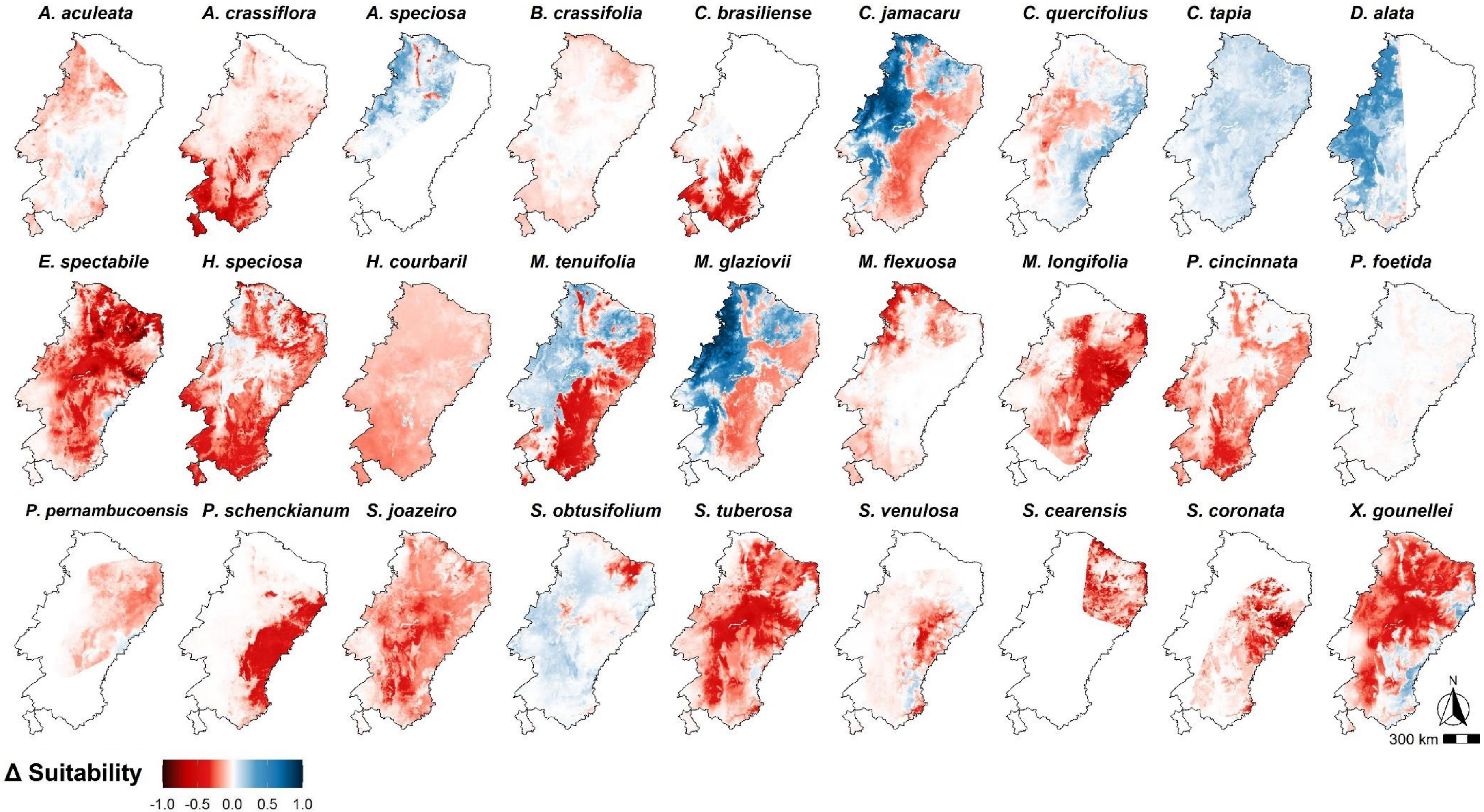

SSP5-8.5 – Cultivated Food Plants

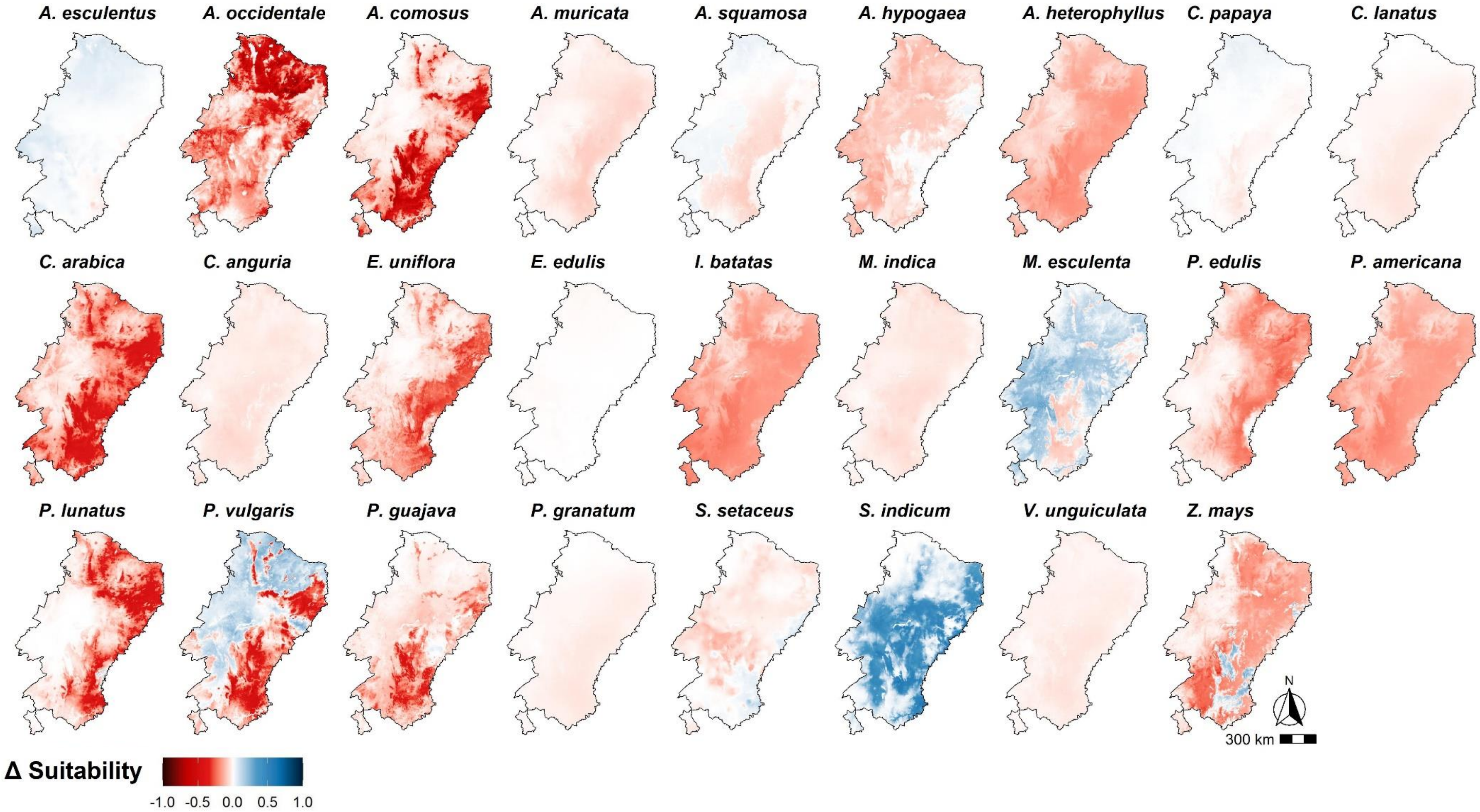

Figure S3. Number of species (cultivated and wild food plants) in the current scenario (1970–2000) and in the four future climate change scenarios (SSPs) (2041–2060) in Brazilian Semiarid.

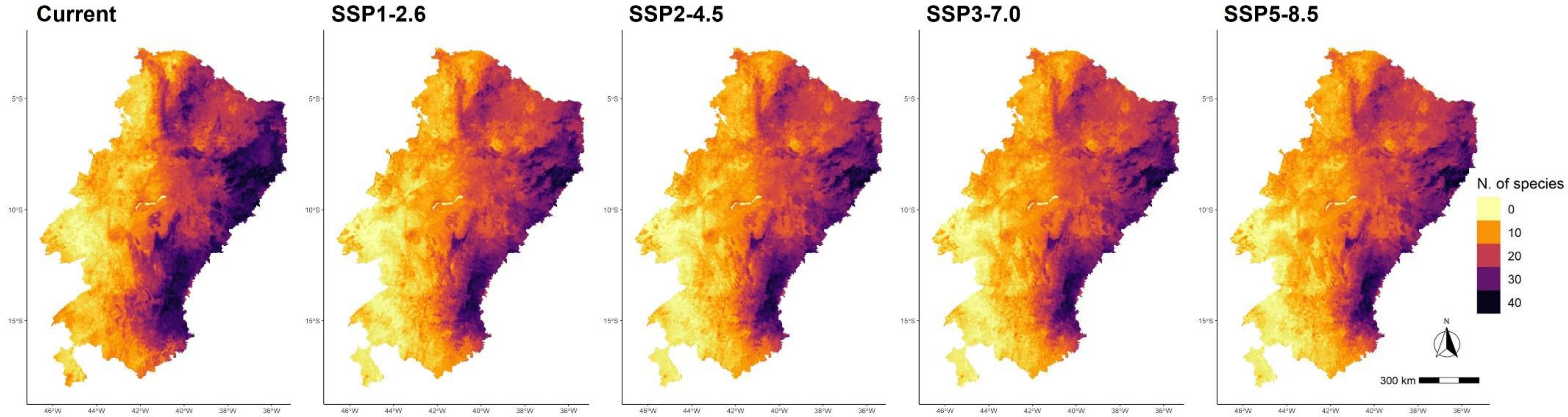

Table S1. Relative contribution (RC) of the yield of cultivated food plants in the Brazilian Semiarid. Data on crop yield (in tons) are from the Brazilian Agricultural Census (IBGE, 2017b, 2017c, 2017d). * Species selected for habitat suitability modeling.

**Species**	**Yield (t)**	**RC (%)**
Zea mays *	604384	21.12348
Manihot esculenta *	505228	17.65794
Saccharum officinarum	308384	10.77815
Cocos nucifera	160933	5.62468
Vigna unguiculata *	145107	5.07155
Citrullus lanatus *	143796	5.02573
Solanum lycopersicum	110991	3.87918
Mangifera indica *	104566	3.65463
Passiflora edulis *	90467	3.16186
Ananas comosus *	84347	2.94796
Cucurbita moschata	59261	2.07120
Phaseolus vulgaris *	58030	2.02817
Anacardium occidentale *	53090	1.85552
Ipomoea batatas *	37018	1.29380
Capsicum annuum	36752	1.28450
Oryza sativa	36627	1.28013
Sicyos edulis	36558	1.27772
Carica papaya *	31398	1.09737
Psidium guajava *	30898	1.07990
Daucus carota	29637	1.03583
Cucumis melo	28334	0.99029
Abelmoschus esculentus *	21505	0.75161
Vitis vinifera	20626	0.72089
Malpighia emarginata	18067	0.63145
Cucumis sativus	13927	0.48675
Phaseolus lunatus *	13393	0.46809
Coffea arabica *	10636	0.37173
Beta vulgaris	10517	0.36757
Artocarpus heterophyllus *	8295	0.28991
Citrus reticulata	8163	0.28530
Annona squamosa *	4764	0.16650
Cucumis anguria *	4753	0.16612
Solanum aethiopicum	4568	0.15965
Solanum melongena	3713	0.12977

Brassica oleracea	3504	0.12247
Theobroma cacao	2991	0.10454
Cucurbita pepo	2868	0.10024
Annona muricata *	2150	0.07514
Fragaria vesca	2120	0.07409
Colocasia esculenta	1740	0.06081
Arachis hypogaea *	1458	0.05096
Dioscorea alata	1301	0.04547
Persea americana *	1130	0.03949
Glycine max	1113	0.03890
Solanum tuberosum	782	0.02733
Arracacia xanthorrhiza	489	0.01709
Plinia peruviana	201	0.00703
Sesamum indicum *	137	0.00479
Selenicereus setaceus *	89	0.00311
Punica granatum *	87	0.00304
Coffea canephora	84	0.00294
Raphanus sativus	67	0.00234
Euterpe oleracea	47	0.00164
Euterpe edulis *	31	0.00108
Brassica rapa	31	0.00108
Elaeis guineensis	9	0.00031
Helianthus annuus	9	0.00031
Paullinia cupana	9	0.00031
Lathyrus oleraceus	7	0.00024
Theobroma grandiflorum	5	0.00017
Secale cereale	2	0.00007
Eugenia uniflora *	1	0.00003
TOTAL	2861195	100.00000

Table S2. Wild and cultivated plants of economic and food importance for the semiarid region of Brazil selected as target species in this study. Taxonomic authorities are in accordance with Plants of the World Online (2022) and Flora e Funga do Brasil (2022). The cultivated plants were initially identified from the database on registered Brazilian cultivars (CultivarWeb, 2022). Abbreviations: IBGE = IBGE database for plants from agriculture and extractive activities in the Brazilian semiarid region (IBGE, 2017a, b, c, d); Jacob = Database for strategic food plants in the Caatinga (Jacob et al., 2020). The numbers represent the references for the taxonomic identification of the species: 1 = Guimarães et al. (2020); 2 = Machado et al. (2015); 3 = Souza et al. (2020); 4 = Mitchell and Daly (2015); 5 = Bailão et al. (2021); 6 = Carvalho (2008); 7 = Porro (2019); 8 = Guimarães et al. (2021); 9 = Takemoto et al. (2001); 10 = San-Martín-Hernández et al. (2023); 11 = Freire Filho (2011); 12 = Faleiro et al. (2005); 13 = Knabben and Costa (2012); 14 = Lovato et al. (2017); 15 = Guedes and Guerra (2007); 16 = Barbosa and Arriel (2019); 17 = Gama (2020); 18 = Mosca et al. (2006).

**Family**	**Scientific name**	**Common name**	**Group**	**Database**
Anacardiaceae R.Br.	*Anacardium occidentale* L.	Caju	Cultivated	IBGE
	Mangifera indica L.	Manga	Cultivated	IBGE
	Spondias tuberosa Arruda	Umbu	Wild	Jacob, IBGE
	Spondias venulosa (Mart. ex Engl.) Engl.	Cajarana	Wild	IBGE (1, 2, 3, 4)
Annonaceae Juss.	*Annona crassiflora* Mart.	Araticum	Wild	IBGE (5)
	Annona muricata L.	Graviola	Cultivated	IBGE
	Annona squamosa L.	Fruta-do-conde	Cultivated	IBGE (18)
Apocynaceae Juss.	*Hancornia speciosa* Gomes	Mangaba	Wild	IBGE (5)
	Mandevilla tenuifolia (J.C.Mikan) Woodson	Manofê	Wild	Jacob
Arecaceae Schultz Sch.	*Acrocomia aculeata* (Jacq.) Lodd. ex R.Keith	Macaúba	Wild	IBGE (6)
	Attalea speciosa Mart.	Babaçu	Wild	IBGE (7)
	Euterpe edulis Mart.	Palmito	Cultivated	IBGE
	Mauritia flexuosa L.f.	Buriti	Wild	IBGE (5)
	Syagrus cearensis Noblick	Coco-catolé	Wild	Jacob
	Syagrus coronata (Mart.) Becc.	Licuri	Wild	IBGE (8)
Bromeliaceae A.Juss.	*Ananas comosus* (L.) Merril	Abacaxi	Cultivated	IBGE
	Encholirium spectabile Mart. ex Schult. & Schult.f.	Macambira-de-flexa	Wild	Jacob
Cactaceae Juss.	*Cereus jamacaru* DC.	Mandacaru	Wild	Jacob
	Pilosocereus pachycladus subsp. *pernambucoensis* (F.Ritter) Zappi	Facheiro	Wild	Jacob
	Selenicereus setaceus (Salm-Dyck) Berg	Pitaia	Cultivated	IBGE
	Xiquexique gounellei (F.A.C.Weber ex K.Schum.) Lavor & Calvente	Xique-xique	Wild	Jacob
Capparaceae A.Juss.	*Crateva tapia* L.	Trapiá	Wild	Jacob
	Morisonia longifolia (Mart.) Christenh. & Byng	Incó	Wild	Jacob
Caricaceae Dumort.	*Carica papaya* L.	Mamão	Cultivated	IBGE
Caryocaraceae Voigt	*Caryocar brasiliense* A.St.-Hil.	Pequi	Wild	IBGE (5)
Convolvulaceae Juss.	*Ipomoea batatas* (L.) Lam.	Batata-doce	Cultivated	IBGE
Cucurbitaceae A.Juss.	*Citrullus lanatus* (Thunb.) Matsum. & Nakai	Melancia	Cultivated	IBGE
	Cucumis anguria L.	Maxixe	Cultivated	IBGE
Euphorbiaceae Juss	*Cnidoscolus quercifolius* Pohl	Favela-branca	Wild	Jacob
	Manihot carthagenensis subsp. *glaziovii* (Müll.Arg.) Allem	Purnunça	Wild	Jacob
	Manihot esculenta Crantz	Mandioca ou aipim	Cultivated	IBGE
Fabaceae Lindl.	*Arachis hypogaea* L.	Amendoim	Cultivated	IBGE
	Dipteryx alata Vogel	Baru	Wild	IBGE (5, 9)
	Hymenaea courbaril L.	Jatobá	Wild	Jacob
	Phaseolus lunatus L.	Fava	Cultivated	IBGE (16, 17)
	Phaseolus vulgaris L.	Feijão	Cultivated	IBGE (13, 14, 15)
	Vigna unguiculata (L.) Walp.	Feijão-fradinho	Cultivated	IBGE (11)
Lauraceae Juss.	*Persea americana* Mill.	Abacate	Cultivated	IBGE
Lythraceae J.St.-Hil.	*Punica granatum* L.	Romã	Cultivated	IBGE
Malpighiaceae Juss.	*Byrsonima crassifolia* (L.) Kunth	Murici	Wild	IBGE (5, 10)
Malvaceae Juss.	*Abelmoschus esculentus* (L.) Moench	Quiabo	Cultivated	IBGE
Moraceae Gaudich.	*Artocarpus heterophyllus* Lam.	Jaca	Cultivated	IBGE
Myrtaceae Juss.	*Eugenia uniflora* L.	Pitanga	Cultivated	IBGE
	Psidium guajava L.	Goiaba	Cultivated	IBGE
	Psidium schenckianum Kiaersk.	Pirim	Wild	Jacob
Passifloraceae Juss. ex Roussel	*Passiflora cincinnata* Mast.	Maracujá-do-mato	Wild	Jacob
	Passiflora edulis Sims	Maracujá	Cultivated	IBGE (12)
	Passiflora foetida L.	Maracujá-de-estralo	Wild	Jacob
Pedaliaceae R.Br.	*Sesamum indicum* L.	Gergelim	Cultivated	IBGE
Poaceae Barnhart	*Zea mays* L.	Milho	Cultivated	IBGE
Rhamnaceae Juss.	*Sarcomphalus joazeiro* (Mart.) Hauenschild	Juá	Wild	Jacob
Rubiaceae Juss.	*Coffea arabica* L.	Café-arábica	Cultivated	IBGE
Sapotaceae Juss.	*Sideroxylon obtusifolium* (Roem. & Schult.) T.D.Penn.	Quixaba	Wild	Jacob

Table S3. Parameters used in habitat suitability models for wild and cultivated food plants. Records = number of occurrence records with coordinates, RM = regularization multiplier (L = linear, Q = quadratic, H = hinge, P = product, T = threshold), FC = resource classes, AICc = corrected Akaike information criterion, AUC = area under the curve receiver operating characteristic values, Threshold = threshold value for binarization (maximum training sensitivity plus specificity). Asterisks represent endemism: * = Species endemic to Brazil; ** = Species endemic to Brazil and restricted to the Caatinga.

Species	Group	Records	RM	FC	AICc	AUC	Threshold	Environmental Variables¹
Abelmoschus esculentus	Cultivated	5	1.5	L	113.88	0.86	0.57	bio_13, bdod_15_30, phh2o_30_60, nitro_0_5, bio_10, bio_02, sand_30_60
Acrocomia aculeata	Wild	441	1	LQHP	11243.46	0.84	0.36	cec_30_60, bio_13, bio_10, ocd_15_30, bio_17, bio_07, sand_30_60, bdod_0_5, nitro_15_30
Anacardium occidentale	Cultivated	110	1	LQHP	2425.37	0.80	0.58	bio_02, sand_30_60, phh2o_30_60, bio_10, bio_13, nitro_0_5, bdod_15_30
Ananas comosus	Cultivated	6	2	L	127.78	0.92	0.36	bio_10, bio_02, bdod_15_30, sand_30_60, nitro_0_5, bio_13
Annona crassiflora	Wild	234	0.5	LQHP	5387.74	0.84	0.43	bio_10, cec_0_5, bio_13, bio_07, ocd_5_15, silt_30_60, bio_14, nitro_15_30
Annona muricata	Cultivated	14	0.5	LQ	319.32	0.82	0.43	bio_02, bio_13, sand_30_60, bdod_15_30, nitro_0_5, bio_10, phh2o_30_60
Annona squamosa	Cultivated	21	1	LQH	490.04	0.80	0.67	bio_02, phh2o_30_60, bdod_15_30, bio_13, sand_30_60
Arachis hypogaea	Cultivated	17	1	LQH	397.10	0.73	0.56	nitro_0_5, bio_02, bio_13, bio_10
Artocarpus heterophyllus	Cultivated	8	0.5	L	180.13	0.80	0.58	bio_02, bio_10, phh2o_30_60, bio_13
Attalea speciosa	Wild	54	1	LQHP	1336.43	0.87	0.52	phh2o_30_60, bio_14, bio_13, bio_06, bio_08, soc_15_30, sand_30_60, nitro_30_60
Byrsonima crassifolia	Wild	2821	0.5	LQHPT	71439.59	0.76	0.46	nitro_30_60, cec_30_60, bio_14, bio_13, bio_08, sand_30_60, bio_07, bdod_0_5, soc_5_15
Carica papaya	Cultivated	23	1.5	LQH	515.18	0.77	0.84	bio_02, phh2o_30_60, nitro_0_5, bdod_15_30, sand_30_60, bio_10, bio_13
Caryocar brasiliense	Wild	121	1	LQH	2756.01	0.84	0.32	bio_08, phh2o_30_60, ocd_5_15, bio_07, silt_30_60, bio_15, soc_15_30
Cereus jamacaru *	Wild	320	1.5	LQHPT	7095.86	0.88	0.31	cec_0_5, bio_07, bio_13, bio_14, sand_30_60, bio_10, silt_30_60, soc_15_30
Citrullus lanatus	Cultivated	7	1	L	158.09	0.80	0.58	bio_02, sand_30_60, phh2o_30_60, bio_10
Cnidoscolus quercifolius *	Wild	265	1	LQHP	5506.04	0.92	0.25	bio_13, nitro_15_30, bio_07, bio_14, bio_10, bdod_0_5, sand_30_60
Coffea arabica	Cultivated	14	1	LQ	302.35	0.83	0.45	bio_10, bio_02, bio_13, sand_30_60, nitro_0_5
Crateva tapia	Wild	1123	0.5	LQHPT	27918.31	0.83	0.35	cec_30_60, bio_08, nitro_30_60, bio_07, bio_14, sand_30_60, bio_13, bdod_0_5
Cucumis anguria	Cultivated	12	2.5	LQ	269.56	0.77	0.55	bio_02, nitro_0_5, bio_13, sand_30_60, phh2o_30_60, bio_10
Dipteryx alata	Wild	359	1	LQHPT	8756.59	0.82	0.56	phh2o_15_30, bio_13, bio_14, silt_0_5, ocd_5_15, nitro_15_30, bio_05, cfvo_15_30, clay_30_60
Encholirium spectabile *	Wild	170	2.5	LQHPT	3673.16	0.86	0.35	cec_0_5, bio_07, bio_13, bio_10, bio_14, sand_30_60
Eugenia uniflora	Cultivated	15	1	LQH	377.39	0.89	0.31	bio_02, nitro_0_5, bio_10, bio_13, phh2o_30_60, bdod_15_30
Euterpe edulis	Cultivated	8	1	L	181.28	0.81	0.55	nitro_0_5, bio_13, bio_10
Hancornia speciosa	Wild	597	0.5	LQHP	14554.26	0.81	0.45	bio_08, bio_13, bio_14, soc_5_15, silt_30_60, ocd_15_30, bio_07, nitro_30_60, cec_5_15
Hymenaea courbaril	Wild	1338	0.5	LQHP	34918.05	0.74	0.53	phh2o_30_60, bio_07, bio_08, bio_14, nitro_30_60, bio_13, sand_30_60, cec_30_60, ocd_15_30
Ipomoea batatas	Cultivated	8	1	L	180.54	0.75	0.65	bio_10, bio_02, nitro_0_5, bio_13
Mandevilla tenuifolia	Wild	586	0.5	LQHP	13480.17	0.86	0.25	bio_05, bio_14, bio_13, bio_07, soc_15_30, nitro_30_60, bdod_15_30, silt_30_60
Mangifera indica	Cultivated	13	0.5	LQ	305.60	0.78	0.47	bio_02, phh2o_30_60, bio_13, sand_30_60, nitro_0_5, bio_10
Manihot carthagenensis *	Wild	94	1.5	LQ	2096.39	0.86	0.36	cec_0_5, ocd_5_15, bio_07, bio_10, silt_30_60, bio_14

Manihot esculenta	Cultivated	69	1	LQH	1548.88	0.76	0.56	bio_02, phh2o_30_60, bio_13, bdod_15_30, sand_30_60, bio_10, nitro_0_5
Mauritia flexuosa	Wild	511	0.5	LQHP	12930.56	0.77	0.50	soc_5_15, bio_13, phh2o_30_60, bio_08, bio_14, bio_07, sand_30_60
Morisonia longifolia *	Wild	366	1	H	7495.74	0.81	0.38	bio_13, bio_02, bio_10, nitro_15_30, silt_30_60
Passiflora cincinnata	Wild	714	0.5	LQHP	17420.78	0.85	0.32	silt_30_60, cec_5_15, bio_08, bio_14, bio_07, ocd_5_15, bdod_15_30
Passiflora edulis	Cultivated	27	1.5	LQH	577.20	0.83	0.65	bio_02, bio_10, bio_13, sand_30_60, nitro_0_5
Passiflora foetida	Wild	2724	0.5	LQHP	70407.64	0.77	0.50	cec_5_15, bio_08, bio_07, bio_14, silt_5_15, bdod_15_30, bio_13, ocd_15_30
Persea americana	Cultivated	9	0.5	L	212.08	0.79	0.68	bio_02, bio_10, sand_30_60, nitro_0_5, bio_13
Phaseolus lunatus	Cultivated	25	1	LQH	533.26	0.90	0.27	bio_02, bio_10, bio_13, sand_30_60, bdod_15_30, nitro_0_5
Phaseolus vulgaris	Cultivated	26	1	LQH	609.56	0.87	0.65	bio_02, bio_10, bdod_15_30, phh2o_30_60, bio_13
Pilosocereus pachycladus **	Wild	67	1	LQ	1318.59	0.80	0.65	bio_14, bio_02, cec_30_60, bio_10, silt_0_5, phh2o_30_60, nitro_15_30
Psidium guajava	Cultivated	57	1	LQH	1259.89	0.82	0.47	phh2o_30_60, bio_02, sand_30_60, bdod_15_30, nitro_0_5, bio_13, bio_10
Psidium schenckianum	Wild	161	1	LQHP	3130.06	0.95	0.15	cec_0_5, bio_10, sand_30_60, bio_14, bdod_5_15, cfvo_30_60, nitro_15_30
Punica granatum	Cultivated	9	0.5	L	213.09	0.75	0.58	bio_02, sand_30_60, bio_13, bio_10
Sarcomphalus joazeiro	Wild	637	0.5	LQHP	14505.81	0.91	0.33	bio_13, cec_15_30, sand_30_60, bio_07, nitro_5_15, bio_14, bio_08, bdod_15_30
Selenicereus setaceus	Cultivated	6	1.5	L	131.78	0.87	0.40	bio_13, bio_02, nitro_0_5, phh2o_30_60, bdod_15_30
Sesamum indicum	Cultivated	6	1	L	160.33	0.96	0.57	bio_13, bio_10, sand_30_60, bio_02, bdod_15_30
Sideroxylon obtusifolium	Wild	605	0.5	LQ	14811.44	0.90	0.22	bio_04, cec_5_15, bio_08, bio_13, bdod_15_30, sand_30_60, silt_30_60, bio_14, ocd_30_60
Spondias tuberosa *	Wild	438	1	LQHP	9507.10	0.89	0.21	phh2o_30_60, bio_13, cec_0_5, bio_07, bio_10, soc_15_30, sand_30_60
Spondias venulosa *	Wild	96	1	H	2012.76	0.94	0.15	bio_14, bio_13, bio_07, bio_04, bdod_15_30, bio_08, silt_30_60
Syagrus cearensis **	Wild	26	1	L	485.83	0.83	0.67	nitro_15_30, bio_10, cec_0_5, bio_14, bio_07, sand_30_60
Syagrus coronata **	Wild	143	2.5	LQHPT	2829.18	0.81	0.42	bio_02, nitro_30_60, bio_13, ocd_30_60, clay_5_15, bio_10, sand_30_60, bdod_30_60
Vigna unguiculata	Cultivated	12	1	LQ	268.58	0.77	0.65	bio_02, sand_30_60, bio_13, phh2o_30_60, bio_10
Xiquexique gounellei *	Wild	232	1	LQHPT	4959.49	0.79	0.40	bio_07, bio_13, bio_14, bio_10, sand_30_60, ocd_30_60
Zea mays	Cultivated	74	1	LQH	1619.44	0.82	0.62	phh2o_30_60, bio_02, bio_13, nitro_0_5, bio_10, sand_30_60, bdod_15_30

[1] **Bioclimatic variables:** bio02 = Mean Diurnal Range (Mean of monthly (max temp - min temp)); bio04 = Temperature Seasonality (standard deviation ×100); bio05 = Max Temperature of Warmest Month; bio06 = Min Temperature of Coldest Month; bio07 = Temperature Annual Range (BIO5-BIO6); bio08 = Mean Temperature of Wettest Quarter; bio10 = Mean Temperature of Warmest Quarter; bio13 = Precipitation of Wettest Month; bio14 = Precipitation of Driest Month; bio15 = Precipitation Seasonality (Coefficient of Variation); bio17 = Precipitation of Driest Quarter. **Soil Properties:** bdod = Bulk density; cec = Cation exchange capacity at ph 7; cfvo = Coarse fragments volumetric; clay = Clay content; nitro = Nitrogen; ocd = Organic carbon densities; phh2o = Soil pH in H2O; sand = Sand content; silt = Silt content; soc = Soil organic carbon content.

Table S4. T-test for the variation in habitat suitability (species) across four future climate change projections (2041-2060). The projections range from a more optimistic scenario to a more pessimistic one regarding greenhouse gas emissions (SSP1-2.6, SSP2-4.5, SSP3-7.0, and SSP5-8.5, respectively). Negative values indicate a reduction in habitat suitability. 95% confidence interval (CI).

T test, Δ Suitability (species), n = 53					
**Scenario**	**Estimate**	**CI low**	**CI high**	**t**	**p**
SSP126	-0.049	-0.068	-0.030	-5.195	< 0.001
SSP245	-0.052	-0.074	-0.030	-4.713	< 0.001
SSP370	-0.055	-0.080	-0.030	-4.461	< 0.001
SSP585	-0.060	-0.088	-0.033	-4.409	< 0.001

Table S5. Variation in habitat suitability values of cultivated and wild species in the Brazilian Semiarid, under current and future climate scenarios (SSPs). Mean, standard deviation (SD) and relative variation of the mean [Δ Mean (%)] of habitat suitability for each species. n = number of pixels that species occupies in the Brazilian Semiarid region, from which the suitability values were extracted.

	Current		SSP1-2.6			SSP2-4.5			SSP3-7.0			SSP5-8.5				
**Species**	Mean	SD	Mean	SD	Δ Mean (%)	Mean	SD	Δ Mean (%)	Mean	SD	Δ Mean (%)	Mean	SD	Δ Mean (%)	n	group
Abelmoschus esculentus	0.39	0.16	0.42	0.17	7.44	0.42	0.17	7.44	0.41	0.16	5.64	0.41	0.17	5.64	53534	cultivated
Acrocomia aculeata	0.26	0.16	0.24	0.14	-8.11	0.24	0.14	-6.56	0.23	0.14	-12.36	0.22	0.14	-13.90	40972	wild
Anacardium occidentale	0.44	0.27	0.30	0.26	-30.66	0.28	0.25	-36.84	0.25	0.24	-43.48	0.22	0.24	-49.89	53534	cultivated
Ananas comosus	0.21	0.24	0.08	0.13	-62.98	0.06	0.11	-69.23	0.05	0.09	-74.52	0.04	0.08	-78.85	53534	cultivated
Annona crassiflora	0.17	0.20	0.07	0.10	-59.04	0.06	0.09	-65.06	0.05	0.08	-69.88	0.04	0.07	-74.70	48962	wild
Annona muricata	0.37	0.26	0.34	0.25	-8.42	0.34	0.25	-7.34	0.34	0.25	-7.88	0.33	0.25	-9.78	53534	cultivated
Annona squamosa	0.50	0.24	0.50	0.24	-1.20	0.50	0.24	-0.80	0.49	0.24	-1.40	0.49	0.24	-2.20	53534	cultivated
Arachis hypogaea	0.56	0.20	0.52	0.19	-8.69	0.51	0.18	-9.04	0.51	0.18	-10.46	0.50	0.18	-11.70	53578	cultivated
Artocarpus heterophyllus	0.46	0.20	0.37	0.18	-19.22	0.37	0.18	-20.95	0.36	0.17	-23.33	0.34	0.17	-25.92	53578	cultivated
Attalea speciosa	0.20	0.24	0.25	0.26	24.75	0.26	0.27	30.81	0.28	0.28	40.40	0.28	0.28	42.93	23581	wild
Byrsonima crassifolia	0.25	0.13	0.24	0.11	-6.30	0.24	0.11	-5.51	0.23	0.11	-9.45	0.23	0.11	-9.84	53534	wild
Carica papaya	0.45	0.24	0.46	0.23	0.44	0.46	0.23	1.32	0.46	0.23	1.10	0.46	0.23	0.66	53534	cultivated
Caryocar brasiliense	0.27	0.27	0.15	0.16	-42.86	0.14	0.15	-46.62	0.13	0.14	-51.13	0.12	0.13	-54.14	24003	wild
Cereus jamacaru	0.48	0.31	0.50	0.28	3.94	0.53	0.28	9.54	0.55	0.28	14.11	0.57	0.28	18.88	53534	wild
Citrullus lanatus	0.54	0.18	0.52	0.18	-3.16	0.52	0.18	-2.60	0.52	0.18	-2.97	0.52	0.18	-3.90	53578	cultivated
Cnidoscolus quercifolius	0.33	0.28	0.33	0.27	-0.90	0.33	0.27	-1.81	0.35	0.27	4.22	0.35	0.27	3.92	53154	wild
Coffea arabica	0.33	0.26	0.19	0.19	-43.77	0.17	0.17	-49.54	0.15	0.16	-55.02	0.13	0.15	-59.88	53578	cultivated
Crateva tapia	0.34	0.21	0.39	0.23	14.53	0.41	0.23	18.90	0.42	0.24	21.80	0.43	0.24	24.42	53534	wild
Cucumis anguria	0.53	0.22	0.50	0.22	-5.84	0.50	0.22	-5.08	0.50	0.22	-5.27	0.50	0.22	-6.40	53578	cultivated
Dipteryx alata	0.32	0.24	0.47	0.25	45.65	0.50	0.25	56.52	0.50	0.26	56.52	0.52	0.26	61.80	30089	wild
Encholirium spectabile	0.44	0.29	0.26	0.26	-40.18	0.24	0.26	-45.89	0.22	0.25	-48.86	0.20	0.24	-54.11	53287	wild
Eugenia uniflora	0.28	0.27	0.20	0.23	-28.98	0.19	0.22	-32.16	0.18	0.21	-36.04	0.17	0.20	-39.93	53534	cultivated
Euterpe edulis	0.53	0.13	0.52	0.13	-0.95	0.52	0.13	-0.95	0.52	0.13	-0.76	0.52	0.13	-0.76	53578	cultivated
Hancornia speciosa	0.46	0.26	0.32	0.19	-29.61	0.31	0.18	-32.02	0.30	0.17	-35.31	0.28	0.17	-38.16	53534	wild
Hymenaea courbaril	0.61	0.16	0.52	0.15	-14.54	0.52	0.15	-15.20	0.51	0.15	-16.83	0.50	0.16	-17.81	53576	wild
Ipomoea batatas	0.55	0.15	0.44	0.14	-19.60	0.43	0.14	-21.96	0.42	0.14	-24.32	0.40	0.13	-26.86	53578	cultivated

Mandevilla tenuifolia	0.39	0.27	0.31	0.18	-20.93	0.31	0.17	-21.19	0.31	0.16	-19.64	0.31	0.16	-19.64	53090	wild
Mangifera indica	0.48	0.24	0.45	0.24	-6.82	0.45	0.24	-6.20	0.46	0.24	-5.99	0.45	0.24	-7.02	53578	cultivated
Manihot carthagenensis	0.49	0.30	0.55	0.29	13.40	0.57	0.30	17.53	0.59	0.30	21.44	0.61	0.30	25.98	53576	wild
Manihot esculenta	0.49	0.24	0.52	0.23	7.41	0.54	0.23	10.70	0.56	0.23	14.81	0.57	0.23	16.87	53534	cultivated
Mauritia flexuosa	0.14	0.14	0.11	0.12	-22.86	0.10	0.11	-26.43	0.09	0.11	-33.57	0.09	0.11	-37.86	53474	wild
Morisonia longifolia	0.36	0.30	0.22	0.25	-37.54	0.20	0.24	-43.42	0.19	0.23	-47.34	0.17	0.22	-53.22	42436	wild
Passiflora cincinnata	0.62	0.24	0.52	0.21	-15.93	0.51	0.21	-16.59	0.51	0.21	-16.91	0.51	0.21	-17.24	53534	wild
Passiflora edulis	0.33	0.29	0.25	0.25	-23.56	0.25	0.25	-25.08	0.24	0.24	-27.49	0.23	0.23	-31.42	53578	cultivated
Passiflora foetida	0.51	0.24	0.50	0.24	-2.53	0.50	0.24	-1.75	0.51	0.24	-0.78	0.51	0.24	-0.39	53534	wild
Persea americana	0.56	0.16	0.46	0.16	-17.77	0.45	0.15	-19.93	0.43	0.15	-22.08	0.42	0.15	-24.42	53578	cultivated
Phaseolus lunatus	0.23	0.27	0.14	0.20	-39.66	0.13	0.18	-43.97	0.12	0.17	-49.57	0.10	0.16	-55.17	53534	cultivated
Phaseolus vulgaris	0.37	0.28	0.32	0.25	-13.39	0.32	0.26	-12.30	0.32	0.25	-12.30	0.32	0.25	-12.57	53534	cultivated
Pilosocereus pachycladus	0.36	0.26	0.30	0.22	-17.22	0.30	0.22	-17.22	0.30	0.22	-18.06	0.29	0.21	-20.83	25753	wild
Psidium guajava	0.46	0.25	0.40	0.23	-13.04	0.40	0.23	-13.70	0.39	0.23	-16.30	0.38	0.23	-17.83	53534	cultivated
Psidium schenckianum	0.16	0.27	0.08	0.16	-50.61	0.07	0.14	-57.32	0.06	0.12	-62.80	0.05	0.11	-67.07	47016	wild
Punica granatum	0.54	0.18	0.52	0.18	-3.33	0.53	0.18	-2.78	0.52	0.18	-3.15	0.52	0.18	-3.89	53578	cultivated
Sarcomphalus joazeiro	0.55	0.31	0.43	0.27	-21.53	0.42	0.27	-23.91	0.42	0.27	-23.91	0.40	0.27	-26.64	53534	wild
Selenicereus setaceus	0.38	0.28	0.34	0.28	-8.27	0.34	0.28	-9.87	0.35	0.28	-6.13	0.35	0.28	-5.87	53534	cultivated
Sesamum indicum	0.19	0.25	0.32	0.32	70.59	0.33	0.32	77.54	0.37	0.34	98.93	0.40	0.35	111.23	53534	cultivated
Sideroxylon obtusifolium	0.48	0.31	0.50	0.30	3.53	0.50	0.30	4.16	0.50	0.30	4.57	0.50	0.30	4.16	53534	wild
Spondias tuberosa	0.46	0.29	0.32	0.26	-30.72	0.30	0.26	-35.73	0.29	0.25	-37.25	0.27	0.24	-41.61	52871	wild
Spondias venulosa	0.18	0.24	0.15	0.22	-17.13	0.15	0.22	-19.34	0.14	0.22	-20.44	0.14	0.22	-23.20	43468	wild
Syagrus cearensis	0.33	0.26	0.20	0.20	-41.27	0.18	0.20	-46.39	0.17	0.19	-50.30	0.15	0.18	-53.92	13186	wild
Syagrus coronata	0.29	0.24	0.23	0.22	-22.26	0.21	0.21	-27.40	0.20	0.21	-32.53	0.18	0.20	-39.38	27957	wild
Vigna unguiculata	0.52	0.22	0.50	0.22	-4.43	0.50	0.22	-3.85	0.50	0.22	-4.05	0.49	0.22	-5.01	53578	cultivated
Xiquexique gounellei	0.46	0.25	0.33	0.23	-28.14	0.31	0.23	-32.47	0.31	0.23	-32.90	0.29	0.22	-37.23	50885	wild
Zea mays	0.41	0.28	0.34	0.25	-18.05	0.33	0.25	-19.02	0.33	0.25	-20.49	0.32	0.24	-23.17	53534	cultivated

Table S6. Results of linear mixed models (LMM) for the variation in habitat suitability values between groups of cultivated (CFP) and wild (WFP) species, in each future scenario (SSPs).Sample number = 53000 (26000 for CFP and 27000 for WFP).

	Estimate	Std. Error	df	t value	Pr(>|t|)
**SSP1-2.6**					
(Intercept)	-0.046	0.001	1665.459	-45.099	0.000
Group Wild	-0.001	0.001	52000.000	-1.472	0.141
**SSP2-4.5**					
(Intercept)	-0.048	0.001	1608.613	-40.500	0.000
Group Wild	0.001	0.001	52000.000	0.968	0.333
**SSP3-7.0**					
(Intercept)	-0.053	0.001	1599.196	-40.271	0.000
Group Wild	0.004	0.001	52000.000	3.142	0.002
**SSP5-8.5**					
(Intercept)	-0.058	0.001	1565.799	-40.107	0.000
Group Wild	0.005	0.001	52000.000	3.952	0.000

References

Bailão EFLC, de Oliveira MG, de Almeida LM, et al (2021) Food Composition Data: Edible Plants in Cerrado. In: Jacob MCM, Albuquerque UP (eds) Local Food Plants of Brazil. Springer International Publishing, Cham, pp 179–224

Barbosa GJ, Arriel NHC (2019) Feijão-fava e a agricultura familiar de Serraria-PB. Cadernos de Ciência & Tecnologia 35:387–403

Carvalho PER (2008) Macaubeira: Acrocomia aculeata. In: Espécies arbóreas brasileiras. Colombo: Embrapa Florestas, Brasília, DF: Embrapa Informação Tecnológica, pp 351–358

CultivarWeb (2022) Registro Nacional de Cultivares. Ministério da Agricultura, Pecuária e Abastecimento. https://sistemas.agricultura.gov.br/snpc/cultivarweb/cultivares_registradas.php? Accessed 2 Apr 2024

Faleiro FG, Junqueira NTV, Braga MF (2005) Maracujá: germoplasma e melhoramento genético. Embrapa Cerrados, 2005., Planaltina, DF

Flora e Funga do Brasil (2022) Jardim Botânico do Rio de Janeiro. http://floradobrasil.jbrj.gov.br/. Accessed 4 Jul 2022

Freire Filho FR (2011) Feijão-caupi no Brasil: Produção, Melhoramento Genético, Avanços e Desafios, 1ª edição. Embrapa Meio-Norte

Gama AT da (2020) Desempenho agronômico, divergência genética, fenotipagem de alta eficiência e qualidade de sementes de variedades crioulas de feijão-fava cultivadas no Semiárido Norte Mineiro. Universidade Federal de Minas Gerais

GBIF.org (2022) Global Biodiversity Information Facility. Free and open access to biodiversity data. https://www.gbif.org/occurrence/search?occurrence_status=present. Accessed 1 Jul 2022

Guedes RE, Guerra JGM (2007) Avaliação de cultivares de Feijão-de-vagem de crescimento determinado sob manejo orgânico, nas condições da Baixada Fluminense - Seropédica/RJ. Embrapa Agrobiologia, Seropédica-RJ

Guimarães ARD, Leão KV, Mapeli AM, Schneider LC (2020) Physical and chemical characterization of cajarana fruits (Spondias dulcis Parkinson). Brazilian Journal of Development 6:6693–6701. https://doi.org/10.34117/bjdv6n2-100

Guimarães J dos S, Shiosaki RK, Mendes MLM (2021) Licuri (Syagrus coronata): características, importâncias, potenciais e perspectivas do pequeno coco do Brasil. Desenvolvimento e Meio Ambiente 58:. https://doi.org/10.5380/dma.v58i0.68852

IBGE (2017a) Censo Agropecuário. Tabela 6949: Quantidade produzida na extração vegetal, por tipologia, produtos da extração vegetal. In: Instituto Brasileiro de Geografia e

Estatística. Sistema IBGE de Recuperação Automática - SIDRA. https://sidra.ibge.gov.br/tabela/6949. Accessed 17 Nov 2022

IBGE (2017b) Censo Agropecuário. Tabela 6953: Quantidade produzida na horticultura, por tipologia, produtos da horticultura. https://sidra.ibge.gov.br/tabela/6953. Accessed 2 Apr 2024

IBGE (2017c) Censo Agropecuário. Tabela 6957: Produção, por tipologia, produtos da lavoura temporária. https://sidra.ibge.gov.br/tabela/6957. Accessed 2 Apr 2024

IBGE (2017d) Censo Agropecuário. Tabela 6955: Produção, por tipologia, produtos da lavoura permanente. https://sidra.ibge.gov.br/tabela/6955. Accessed 2 Apr 2024

Jacob MCM, Medeiros MFA de, Albuquerque UP (2020) Biodiverse food plants in the semiarid region of Brazil have unknown potential: A systematic review. PLOS ONE 15:e0230936. https://doi.org/10.1371/journal.pone.0230936

Knabben CC, Costa JS (2012) Manual de classificação do feijão: instrução normativa n. 12, de 28 de março de 2008. Brasília, DF: Embrapa, 2012.

Lovato F, Kowaleski J, Silva SZ da, Heldt LFS (2017) Composição centesimal e conteúdo mineral de diferentes cultivares de feijão biorfortificado (Phaseolus vulgaris L.). Braz J Food Technol 21:. https://doi.org/10.1590/1981-6723.6817

Machado M, Carvalho P, van den Berg C (2015) Domestication, hybridization, speciation, and the origins of an economically important tree crop of Spondias (Anacardiaceae) from the Brazilian Caatinga dry forest. Neodiversity 8:8–49. https://doi.org/10.13102/neod.81.2

Mitchell JD, Daly DC (2015) A revision of Spondias L. (Anacardiaceae) in the Neotropics. PhytoKeys 55:1–92. https://doi.org/10.3897/phytokeys.55.8489

Mosca JL, Cavalcante CEB, Dantas TM (2006) Características botânicas das principais anonáceas e aspectos fisiológicos de maturação. Embrapa Agroindústria Tropical. ISSN 1677-1915, Fortaleza, CE

Porro R (2019) A economia invisível do babaçu e sua importância para meios de vida em comunidades agroextrativistas. Bol Mus Para Emílio Goeldi Ciênc hum 14:169–188

POWO (2022) Plants of the World Online. In: Facilitated by the Royal Botanic Gardens, Kew. http://www.plantsoftheworldonline.org/. Accessed 4 Jul 2022

San-Martín-Hernández C, Martínez-Téllez M, Valenzuela-Amavizca O, et al (2023) Byrsonima Crassifolia L. Kunth a Bio-Resource with Potential: Overview and Opportunities. Folia Horticulturae. https://doi.org/10.2478/fhort-2023-0005

Souza FX de, Porto Filho F de Q, Mendes NVB (2020) Umbu-cajazeira: descrição e técnicas de cultivo. EdUFERSA, Mossoró, RN

Takemoto E, Okada IA, Garbelotti ML, et al (2001) Composição química da semente e do óleo de baru (Dipteryx alata Vog.) nativo do Município de Pirenópolis, Estado de

Goiás. Revista do Instituto Adolfo Lutz 60:113–117.
https://doi.org/10.53393/rial.2001.v60.35540
